# Risk factors for acute abdominal pain (colic) in the adult horse: A scoping review of risk factors, and a systematic review of the effect of management-related changes

**DOI:** 10.1371/journal.pone.0219307

**Published:** 2019-07-11

**Authors:** Laila Curtis, John H. Burford, Gary C. W. England, Sarah L. Freeman

**Affiliations:** School of Veterinary Medicine and Science, University of Nottingham, Sutton Bonnington, Loughborough, Leicestershire, United Kingdom; University of Illinois, UNITED STATES

## Abstract

Acute abdominal pain (colic) is the most common reason for emergency veterinary treatment in the horse. Consolidation of data through a systematic review is important to inform evidence-based medicine and clinical guidelines, but there are currently no published systematic reviews on colic in the horse. The aim of this study was to identify, categorize and appraise the evidence on factors associated with increased risk of developing abdominal pain (colic) due to gastrointestinal disease in the adult horse. A scoping review was performed to identify and categorize evidence on all risk factors for colic. A systematic review of management-related risk factors was then performed following PRISMA guidelines. Both searches were conducted in Medline, CAB Abstracts and Web of Science databases, and publications were assessed against inclusion and exclusion criteria. For the scoping review, study and participant characteristics of included publications and key results were extracted and tabulated. For the systematic review, cohort, case-control or cross-sectional studies investigating acute abdominal pain in horses within two weeks of management changes were assessed. Study characteristics, participant characteristics and study results of included publications for the systematic review were extracted and tabulated. Included publications were appraised using the Joanna Briggs Institute Critical Appraisal Tools for cohort, case-control and cross-sectional studies. The scoping review search identified 3,756 publications. Fifty eight studies met final inclusion criteria, and 22 categories of risk factors were identified. These were grouped into three broad areas: horse-related factors, management-related factors and environment-related factors. The largest body of evidence related to management change. The systematic review of management change identified 410 publications: 14 met inclusion criteria for analysis. These consisted of one cohort, eight case-control and five cross-sectional studies. The studies were conducted between 1990–2008, and the majority of studies were located in the USA (8/14) or UK (3/14). The risk factors related to management change that were assessed were feed, carer, exercise, pasture, water and housing. The largest bodies of evidence for increased risk of colic associated with management change were changes in feed (5/14 publications) and recent change in housing (3/14). Most studies (8/14) did not meet the JBI criterion on confounding factors. There was marked heterogeneity of study methodologies and measures. This is the first study to use a combined scoping and systematic review to analyse evidence for modifiable risk factors for a common condition in the horse. It provides a comprehensive review that will be a key resource for researchers, veterinary practitioners and horse owners. It identified modifiable risk factors associated with an increased risk of colic which should be a key target for preventative health programmes. The findings from the critical appraisal were used to develop recommendations for future research to improve the quality of evidence-based veterinary medicine.

## Introduction

The term ‘colic’ is used to describe abdominal pain in the horse [1]. It can be caused by a range of different diseases affecting the abdominal organs, but acute gastrointestinal disease is the most common reason for horses showing signs of colic [2]. Colic is the most common reason for emergency veterinary treatment [3], and a major reason for death or euthanasia across a range of international studies [4–6]. Recent research has shown that approximately one fifth of colic cases that presented in primary practice are critical (requiring intensive medical care, surgery, euthanasia or that result in death), and up to 16% of cases that present with colic are euthanased or die [2], highlighting that colic is a major health and welfare concern in the horse.

Understanding the factors associated with an increased risk of horses developing abdominal pain is important for both horse owners and veterinary surgeons; evidence on risk factors can help identify animals at increased risk, and inform management strategies to reduce or prevent disease. There have been many attempts to identify risk factors for abdominal pain, and these are represented by a wide and diverse range of publications using a range of approaches. Some studies have investigated factors associated with abdominal pain caused by a range of different diseases [1, 7, 8], whilst others have investigated factors associated with specific diseases causing clinical signs of abdominal pain [9–11]. Currently there are narrative reviews of risk factors for colic [12, 13], but no published systematic reviews in this area. Consolidation of evidence through a systematic review is important to identify the best-evidence available, highlight gaps in the current research [14], and contribute to evidence-based guidelines to assist horse owners and veterinary surgeons. Scoping reviews are essential where there is a large and diverse evidence base, to provide a broad overview of the current evidence, and identify areas suitable for more detailed evaluation in a systematic review [15]. There are a range of different frameworks which have been developed to optimise the process of systematic reviews. PRISMA (Preferred Reporting Items for Systematic Reviews and Meta-Analyses) is widely accepted as the methodological framework for systematic reviews, and is recommended by many journals. PRISMA provides an evidence-based minimum set of items that should be evaluated and reported, and their resources include a standardised checklist and flow diagram [16]. In addition to this, there are large organisations / collaborative groups which both conduct systematic reviews, and provide detailed methodological information and training on performing systematic reviews. Cochrane is a global network developed to promote evidence synthesis, systematic reviews and promoted evidence-based decisions in human medicine [14]. The Cochrane Systematic Reviews are probably the most well recognised collection of systematic reviews in healthcare worldwide. There are a number of other organisations that do similar work, sharing methodology, providing training and collating systematic reviews, including the Joanna Briggs Institute (JBI). The JBI resources include a range of critical appraisal tools for different study designs, to enable individual studies to be evaluated [17]. The common goal of all the organisations is to develop high quality evidence to underpin clinical decision-making.

The aim of the scoping review was to systematically identify and map the current evidence on factors associated with the development of abdominal pain associated with gastrointestinal disease in the adult horse.

The objectives of the scoping review were:

To identify the currently available, published, peer-reviewed literature on risk factors for abdominal pain (colic) in adult horses through a systematic search of databases,

To extract data on study and participant characteristics from included publications to categorise key themes and findings and identify bodies of evidence suitable for future systematic review/s.

The outcomes of the scoping review were used to inform the risk factors that were investigated in detail in the systematic review.

The aim of the systematic review was to appraise current evidence on the association between management-related factors and risk of developing abdominal pain associated with gastrointestinal disease in adult horses, compared to horses that have not been exposed to a management-related factor.

The objectives of the systematic review were:

To identify the currently available, published, peer-reviewed literature on management-related factors associated with the risk of developing abdominal pain in adult horses through a systematic search of databases,

To evaluate the quality of evidence on management-related factors associated with the risk of developing abdominal pain using the Joanna Briggs Institute-Mastari Tools,

To summarise the evidence on management-related risk factors for abdominal pain to develop recommendations on preventative measures and future research.

## Materials and methods

### Protocol and registration

The scoping review adheres to The Joanna Briggs Institute (JBI) systematic scoping review protocol guidelines [17] in addition to findings by Tricco *et al*. [18]. The systematic review adheres to PRISMA guidelines (S1 Checklist). Neither review protocols were registered externally. Protocols for both the scoping review and systematic reviews were developed prior to data extraction (S1 Protocol and S2 Protocol, respectively).

### Search strategy

The databases used for the scoping reviews were:

Medline In-Process & Non-Indexed Citations and Ovid MEDLINE: 1946—present

CAB Abstracts (Ovid): 1910 –present

WEB of Science (Core Collection: Citation Indexes): 1950 –present

The search terms used for both reviews are described in the protocols (S1 Protocol and S2 Protocol).

### Study selection

A primary literature search of databases for the scoping review was conducted between 23–26.11.12, using the search terms described, and then repeated on 23.04.18. The results from 23.04.18 only are presented in this paper. A primary literature search of the databases for the systematic review was conducted on 29.1.18. The results of each search were downloaded into bibliological software EndNote X6 (Thomson Reuters). Duplicates were searched for by author, title and reference and the least complete citation of each duplicate was deleted within EndNote after each database search and extraction was complete. Publications were then assessed through three stages: review of titles for suitable publications, review of abstracts against inclusion and exclusion criteria, and review of the full publications. All titles within the EndNote library were examined, and their abstracts reviewed. Ambiguous titles were retained for further review at the next stage (review of abstract) (S1 Checklist).

Abstracts from these publications were independently assessed by two researchers (SF and LC), for agreement with inclusion and exclusion criteria (Tables 1 and 2). Any ambiguous publications were retained and reviewed in the next step (review of the full publication). The full text of the final publications were independently assessed by two researchers (SF and LC) to confirm eligibility for this review (S1 Checklist).

**Table 1:** Inclusion and exclusion criteria for a scoping review of risk factors associated with the development of abdominal pain (colic) in horses and ponies.

Criteria	Inclusion	Exclusion
**Population**	All types of domesticated equids (horses and ponies)	Donkeys or mules, non-equids, foals/neonates
**Concept**	Development of any clinical signs of colic/abdominal pain as recognised by owner/carer or veterinary surgeon, irrespective of severity or survival outcome Abdominal pain relating to diseases of the gastrointestinal tract Single and recurrent episodes of abdominal pain Abdominal pain occurring >30 days following abdominal surgery	Abdominal pain arising from non-gastrointestinal causes Abdominal pain occurring <30 days following abdominal surgery
**Context**	All languages if translation available Publications investigating diagnostic test/s in order to identify a potential risk factor for colic	Translation not available Publications investigating prognostic and/or diagnostic test/s in order to diagnose a disease or clinical sign relating to colic Studies of treatment/s for colic Studies seeking to establish pain scores for colic
**Study design**	Cohort, case-control or cross-sectional studies	Case series, case reports, randomised controlled trials, narrative reviews, textbook chapters
**Publication type**	Peer and non-peer reviewed publications Research presented in conference proceedings Studies published post-1960	Unable to obtain full study details Studies published pre-1960

**Table 2:** Inclusion and exclusion criteria for a systematic review of management-related factors associated with the risk of developing abdominal pain (colic) in adult horses.

Criteria	Inclusion	Exclusion
**Population**	All types of domesticated equids (horses and ponies)	Donkeys or mules, non equids, foals/neonates
**Exposures**	Change in management (feeding frequency and type, housing, pasture access or exercise) in 2 weeks prior to assessment	No mention of management change
**Comparator**	No change in management (feeding frequency and type, housing, pasture access or exercise) in 2 weeks prior to assessment	
**Outcome**	Development of any clinical signs of colic / abdominal pain as recognised by owner/carer or veterinary surgeon, irrespective of severity or survival outcome Abdominal pain relating to diseases of the gastrointestinal tract Single and recurrent episodes of abdominal pain Abdominal pain occurring >30 days following abdominal surgery	Abdominal pain arising from non-gastrointestinal causes Publications which related to specific diseases causing clinical signs of abdominal pain for example grass sickness, lipoma or enterolithiasis Abdominal pain occurring <30 days following abdominal surgery
**Language**	All languages if translation available	Translation not available
**Study design**	Cohort, case-control or cross-sectional studies	Case series, case reports, randomised controlled trials, narrative reviews, textbook chapters
**Publication type**	Peer and non-peer reviewed publications Research presented in conference proceedings	Unable to obtain full study details

### Eligibility criteria

The inclusion and exclusion criteria for the scoping review is described in Table 1. A new case of abdominal pain was described as such if onset occurred at least seven days after the end of the previous episode [19]. A study was included if the full text could be obtained from any of the University of Nottingham libraries or e-libraries, through University of Nottingham journal subscriptions, during one of three visits to the British Library, or from free online Open Access. In order to determine study design, published definitions were used [20–22]

### Charting process for the scoping review

#### Data collection process

The primary researcher received formal (taught graduate programme) and informal (group and individual discussions) in systematic review methodology. To ensure a common methodological approach and identify any areas which required further clarification, both researchers reviewed together and discussed three of the systematic review papers using the Joanna Briggs Institute (JBI) Critical Appraisal tools [17], prior to performing independent analysis of all papers. The final publications were independently examined by two reviewers (LC and SF). For each JBI tool criterion, publications were rated either ‘Yes’, ‘No’, ‘Unclear’ or ‘Not Applicable’. Any disagreements that arose between the reviewers were resolved through discussion with a third reviewer (JB). Outcomes of this quality assessment were used to generate a summary of the critical appraisal of each study. Meta-analysis was not performed due to heterogeneity in methodology of the publications.

#### Data extraction

Study and participant characteristics of included publications for the scoping review were extracted and tabulated along with a separate table of key results and a summary of findings. Information collected from each publication included author, country of origin, study aims/purpose, study design, how colic was diagnosed, whether surgery/necropsy was used to confirm cases, trial sample size, number of horses with colic, study population, risk factors assessed by multivariable analysis and results.

Study characteristics, participant characteristics and study results of included publications for the systematic review were extracted and presented. Information collected from each publication included study date, design, how colic was diagnosed, whether surgery/necropsy was used to confirm cases, study population, trial sample size, number of horses with colic, which management factors were assessed and funding sources.

#### Quality appraisal and risk of bias for the systematic review

Methodological quality or risk of bias of included studies for the scoping review was not appraised, consistent with guidance on scoping review conduct [17, 18].

Cohort, case-control and cross-sectional studies for the systematic review were appraised against the Joanna Briggs Institute Critical Appraisal tools appropriate for each study design.

#### Synthesis of results for the systematic review

Summary measures used by each publication in the systematic review were recorded. The methodological features of all publications were extracted and an evidence summary presented for each study.

#### Additional analyses

No additional analyses were conducted.

## Results Part 1. Scoping review of all risk factors

### Study selection

**Fig 1:**
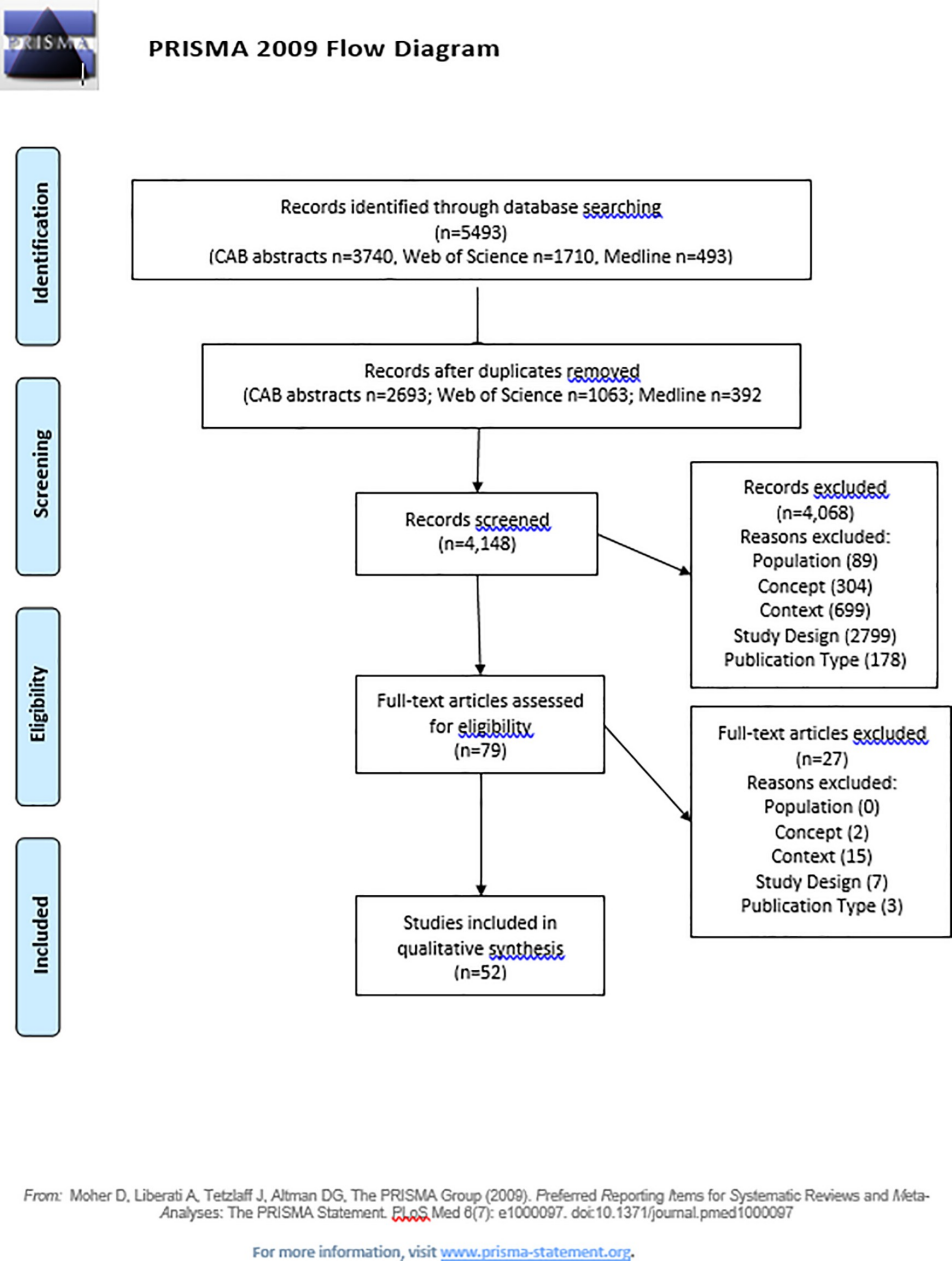
PRISMA 2009 flow diagram for the numbers of studies identified, screened, assessed for eligibility, and included in a scoping review of the risk factors for colic.

The initial search identified 5,943 publications; 3,756 publications remained following review of the titles and removal of duplicated publications. These abstracts were reviewed against inclusion and exclusion criteria. Full text review was performed on 79 publications; a total of 52 studies continued through to the final charting process (Fig 1).

### Study characteristics

The 52 included studies were published between 1989–2017. The majority (38/52) were published in or after the year 2000, with nine studies published in or after 2014.

Of the 52 included publications on risk factors for colic, four studies were conducted across populations of horses based in more than one country and the remaining 48 were based in a single country. There were 19 based in the USA, 16 in the UK, two studies were based in Sweden, two were based in Iran, and the remainder of the publications consisted of one study each conducted across a range of countries (Albania, Austria, Canada, Denmark, Greece, Egypt, Italy, Netherlands, Nigeria) (Table 3). The most commonly used study design was case-control studies (33/52 publications) and cross–sectional studies (11/52 publications); there were four retrospective cohort studies and four prospective cohort studies (Table 3).

**Table 3:** Study characteristics for 52 publications identified in a scoping review for risk factors for colic in the horse.

Author (Year)	Country of origin	Aims/Purpose	Study design*	Colic diagnosis*	Cases confirmed on surgery/ necropsy	Trial sample size (No. with colic of interest)	Study population	Risk factor/s assessed by multivariable analysis
Archer *et al*. (2014) [23]	UK	To investigate temporal changes in IFEE (idiopathic focal eosinophilic enteritis) risk	CC	VS	Yes	850 colic (85 IFEE)	Equine hospital	Age, time, season, geographical location
Archer *et al*. (2008) [6]	UK, USA, Ireland	To identify horse/management risk factors for EFE (epiploic foramen entrapment)	CC	VS	Yes	310 (119 EFE)	University and private clinic	Behaviour, previous colic, carer, height
Archer *et al*. (2008) [9]	UK	To identify horse/management risk factors for EFE and explore seasonality	CC	VS	Yes	293 (77 EFE)	University and private clinic	Behaviour, previous colic, housing, feeding practice
Archer *et al*. (2006) [24]	UK	To determine evidence of seasonality with particular types of colic	CC	VS	Some	2580 (2580)	Referral hospital	Season
Archer *et al*. (2004) [25]	UK, USA	To investigate an association between crib-biting and EFE	CC	VS	Yes	789 (68)	Referral hospital	Crib biting behaviour
Archer *et al*. (2004) [26]	UK	To identify risk factors for EFE	CC	VS	Yes	1350 (71)	Referral hospital	Breed, behaviour, season
Back *et al*. (2013) [27]	Sweden	To investigate A*noplocephala perfoliata* as a risk factor for colic	CC	VS	No	134 (67)	Referral hospital	A*noplocephala perfoliata* infection in faeces
Bizhga *et al*. (2017) [28]	Albania	To identify risk factors for colic	XS	VS	Some	68 (68)	General practice	No significant associations found for increased risk of colic using multivariable analysis
Boswinkel *et al*. (2007) [29]	Netherlands	To determine the importance of *Anoplocephala perfoliata* in horses with colic	CC	VS	Varied between groups	320 (171)	University teaching hospital	Serum A*noplocephala perfoliata* antibody levels
Cohen *et al*. (2006) [30]	USA	To determine whether feeding practices increases risk of DPJ (duodenitis-proximal jejunitis)	CC	VS	No	331 (70)	University teaching hospital	Gender, weight, feed amount, turnout
Cohen *et al*. (2000) [31]	USA	To identify risk factors for enterolithiasis	CC	VS	Yes	130 (26)	University teaching hospital	Feed, time outdoors, breed
Cohen *et al*. (1999) [32]	USA	To determine whether dietary or other management factors are associated with colic	CC	VS	No	2060 (1030)	Multi-practice	Housing, history, season, feeding practices, anthelmintics, breed, activity, age
Cohen and Peloso (1996) [33]	USA	To identify risk factors for recurrent and chronic, intermittent colic	CC	VS	No	1642 (821)	Multi-practice	History, age, feeding practices, housing, breed
Cohen *et al*. (1995) [8]	USA	To determine whether husbandry or health management factors are associated with colic	CC	VS	No	1642 (821)	Multi-practice	History, feeding practices, housing, exercise
Diakakis and Tyrnenopoulou (2017) [34]	Greece	To evaluate the possible correlation between relative humidity and temperature changes and colic	CC	Unclear	No	823 (245)	General practice	No significant associations found for increased risk of colic using multivariable analysis
Egenvall *et al*. (2008) [35]	Sweden	To describe the occurrence of colic, as defined by veterinary insurance claims and risk factors in primary care for colic.	RCo	VS	Unclear	116,288 (3100)	Insured horses	No significant associations found for increased risk of colic using multivariable analysis
Escalona *et al*. (2014) [36]	UK	To determine the pre- valence of colic in a population of crib-biting and/or windsucking horses and to identify horse- and management-level risk factors for colic.	XS	VS/C	No	367 (130)	Horses with crib-biting/windsucking behaviour	Duration of ownership, behaviour, housing, turnout, routine healthcare
Hassanpour *et al*. (2008) [37]	Iran	To identify risk factors for colic	XS	Unclear	No	260 (23)	Equine farms	Housing, pasture, type of feedstuffs, nutrition, events
Hassel *et al*. (2008) [38]	USA	To evaluate dietary and environmental risk factors for colic	CC	VS	Some	136 (61)	University teaching hospital	Breed, feed, housing
Hassel *et al*. (2004) [39]	USA	To identify risk factors for occurrence of colic and improve understanding of the dis- ease pathogenesis	CC	VS	Yes	62 (43)	University teaching hospital	Feed, turnout
Hillyer *et al*. (2002) [40]	UK	To investigate risk factors for simple colonic obstruction and distension in comparison to the general horse population	CC	VS	Some	227 (76)	University teaching hospitals	Behaviour, turnout, exercise, anthelmintic, transport
Hillyer *et al*. (2001) [19]	UK	To estimate the incidence of colic, seasonal pattern, outcome of colic episodes and any association between premises level variables and colic.	XS	VS/C	Unclear	7757 (509)	Thoroughbred training premises	No significant associations found for increased risk of colic using multivariable analysis
Hudson *et al*. (2001) [41]	USA	To determine whether specific feeding practices were associated with development of colic.	CC	VS	Unclear	364 (182)	General practice	Feed, pasture, water and anthelmintics
Husted *et al*. (2005) [10]	Denmark	To investigate the influence of soil type on the risk of ingestion of sand.	RCo	Unclear	No	211 (119)	Stud yards	No significant associations found for increased risk of colic using multivariable analysis
Kaneene *et al*. (1997) [7]	USA	To describe the occurrence of colic and to evaluate associations of selected risk factors with the development of colic.	XS	VS/C	Some	3175 (62)	Equine farms	Housing, use, feeding, watering, anthelmintics
Kaya *et al*. (2009) [42]	Austria	To determine possible alterable and non-alterable risk factors of equine colic in Austria	CC	VS	Unclear	2743 (366)	University teaching hospital	Gender, breed, housing, use, watering, anthelmintics
Leblond *et al*. (2002) [43]	Belgium, France, Germany Switzerland, UK	To assess the importance of colic as a cause of death and to evaluate digestive parasitism as a risk factor for death from colic	CC	VS	Yes	842 (421)	Post-mortem horses	Age, gender, parasitic lesions, breed
Little and Blikslager (2002) [44]	USA	To determine if horses fed Coastal Bermuda grass hay are at risk for development of ileal impaction and if horses that were not treated with any pyrantel salt in the 3 months prior to admission were also at risk.	CC	VS	Yes	278 (78)	University teaching hospital	Feed, anthelmintics
Malamed *et al*. (2010) [45]	USA	To investigate the relationship between crib-biting/windsucking, behaviour and colic	CC	VS	No	574 (347)	University teaching hospital	No significant associations found for increased risk of colic using multivariable analysis
Mehdi and Mohammad (2006) [1]	Iran	To evaluate the frequency of colic, the number of deaths, associated risk factors, and economic loss due to colic.	XS	VS	No	128 (128)	Race and endurance yards	No significant associations found for increased risk of colic using multivariable analysis
Morris *et al*. (1993) [46]	USA	To identify signalement and management factors associated with specific causes of colic.	XS	VS	Some, but numbers not given	449 (449)	University teaching hospital	Chi–squared analysis–significant difference between age, gender, breed, feeding and anthelmintic between different types of colic
Morris *et al*. (1989) [47]	USA	To determine if age, sex, breed, management and history differed between colic cases	XS	VS	Some	1937 (229)	University teaching hospital	No significant associations found for increased risk of colic using multivariable analysis
Olusa (2014) [48]	Nigeria	To investigate if dental abnormalities and lack of routine dental care could predispose horses to colic	CC	Unclear	Unclear	144 (74)	Polo club	No significant associations found for increased risk of colic using multivariable analysis
Patipa *et al*. (2012) [49]	USA	To examine the incidence of colic in equids hospitalised for treatment of ocular disease and to identify risk factors associated with colic in this population	RCo	VS	Some	337 (72)	University teaching hospital	Age, hospitalisation time
Proudman and Holdstock (2000) [50]	UK	To identify if risk of ileal impaction and spasmodic colic increases with *Anoplocephala perfoliata* infection intensity.	CC	Unclear	No	27 (13)	Training and rehabilitation yard yard	No significant associations found for increased risk of colic using multivariable analysis
Proudman *et al*. (1998) [51]	UK	To identify an association between *Anoplocephala perfoliata* and colic	CC	VS	Some	266 (123)	Multi-practice	Tapeworm infection intensity
Proudman and Edwards (1993) [52]	UK	To identify an association between *Anoplocephala perfoliata* and colic	CC	VS	Some	231 (116)	University teaching hospital	No significant associations found for increased risk of colic using multivariable analysis
Proudman (1991) [53]	UK	To quantify types of colic in general practice and their risk factors, to record seasonal incidence and establish any correlation with weather changes, to identify risk factors for spasmodic colic	CC	VS	Some	279 (179)	General practice	No significant associations found for increased risk of colic using multivariable analysis
Reeves *et al*. (1996) [54]	USA & Canada	To identify risk factors for acute equine colic, and generate new hypotheses regarding plausible causal relationships for the syndrome	CC	VS	Unclear	812 (406)	Multi-practice	Housing, age, carer
Reeves *et al*. (1989) [55]	USA	To compare age, sex and breed of colic horses vs controls, to evaluate the influence of these factors on the frequency of surgical and medical treatments and overall surgical survival rate, to report the relative frequency of diagnoses and associated survival rates	CC	VS	Some	3924 (314)	University teaching hospital	No significant associations found for increased risk of colic using multivariable analysis
Salem *et al*. (2017) [56]	Egypt	To determine the prevalence of, and risk factors for colic in a working horse population in Egypt and to describe management practices	XS	O/C	No	342 (191)	Working horses	Dental concerns, behaviour, feed, anthelmintics, coprophagia
Scantlebury *et al*. (2015) [57]	UK	To identify risk factors for recurrent colic (including those factors which may vary over time) among the veterinary-accessing general horse population	CC	VS/C	No	236 (59)	Multi-practice	Behaviour, turnout, feed, probiotics
Scantlebury *et al*. (2011) [58]	UK	To determine the incidence rate of and risk factors for recurrent colic	PCo	VS/C	No	127 (127)	Multi-practice	Dental problem, behaviour
Scherrer *et al*. (2016) [59]	USA	To determine interval prevalence of and factors associated with colic in horses hospitalised for ocular/orthopaedic disease.	XS	VS	No	302 (17)	University teaching hospital	Age, medication, disease type, gender, hospital procedure, antimicrobial use
Senior *et al*. (2004) [60]	UK	To estimate the prevalence of, and identify the risk factors for development of colic in horses after surgery.	RCo	VS	No	428 (14)	University teaching hospital	Opioid use, out of hours cases
Stancampiano *et al*. (2017) [61]	Italy	To compare parasitological status between subjects with or without colic, with particular attention to small strongyle infections	XS	VS	No	86 (43)	University teaching hospital	Positivity to cyathostomine and *S*. *vulgaris*
Suthers *et al*. (2013) [62]	UK	To investigate risk factors for large colon volvulus in the horse	CC	VS	Yes	279 (63)	Multi-practice	Parity, height, carer, premises, stabling, medication, quidding, turnout, feed, hospital
Tinker *et al*. (1997) [63]	USA	To identify risk factors for colic	PCo	VS/C	No	1427 (86)	31 horse farms	Age, history, feed, vaccination
Tinker *et al*. (1997) [5]	USA	To estimate the incidence and mortality rate of colic, frequency of colic and evaluate risk factors.	PCo	VS/C	No	1427 (86)	31 horse farms	No significant associations found for increased risk of colic using multivariable analysis
Traub-Dargatz *et al*. (2001) [4]	USA	To estimate the national incidence of, operation-level risk factors for, and annual economic impact of colic among horses in the United States	PCo	VS	No	21,820 (Unclear)	National Animal Health Monitoring System data	No significant associations found for increased risk of colic using multivariable analysis
Trotz-Williams *et al*. (2008) [64]	Canada	To investigate whether there is an association between infection with *A*. *perfoliata* and risk of colic in horses in Ontario, and identifying potential risk factors for exposure to A. perfoliata.	CC	VS	No	234 (117)	Multi-practice	No significant associations found for increased risk of colic using multivariable analysis
Uhlinger (1990) [65]	UK	To evaluate the effect of anthelmintic schedules on the incidence of colic	CC cross- over	VS	No	Approx. 156 (Unclear)	Privately owned herds	No significant associations found for increased risk of colic using multivariable analysis

* VS = Veterinary practitioner—physical examination, diagnostic tests, or surgery or necropsy. VS/C = Veterinary practitioner and/or carer of the horse. O/C = Horse owner and/or carer. Co = Cohort, CC = Case-control, XS = Cross-sectional, RCo = Retrospective cohort, PCo = Prospective cohort

Twelve of the 52 studies specified that they aimed to investigate risk factors associated with specific types of colic (idiopathic focal eosinophilic enteritis, epiploic foramen entrapment, duodenitis-proximal jejunitis, sand colic, enterolithiasis, ileal impaction, spasmodic colic, simple colonic obstruction and displacement, and colon volvulus). Three studies aimed to investigate risk factors associated with recurrent colic. The remaining 37 studies had aims relating to risk factors associated with colic across a range of different causes/diseases (Table 3).

The diagnosis of colic was made by a veterinary surgeon in the majority of studies (38/52 publications), by the veterinary surgeon or carer in seven studies and the owner/carer in one study. The person who made the diagnosis was unclear or the information was not provided in five studies (Table 3). Confirmation of the diagnosis on necropsy/surgery varied: ten of the 52 studies confirmed diagnosis on surgery/necropsy, 13 confirmed diagnosis on surgery/necropsy in some cases, 23 studies did not confirm diagnosis on surgery/necropsy, and in six studies this was unclear or not the information was not presented (Table 3).

The majority of studies (25/52) were conducted in hospital populations (University teaching / private referral hospitals), 13 studies were conducted in general practice / multi practice populations, and eight studies in farms / herds / yard populations. The remaining studies were conducted in specific populations (e.g. horses that showed crib-biting behaviour, insured horses in Sweden, working equids in Egypt) (Table 3).

A wide variety of potential risk factors were investigated and further details are provided on these in Table 4.

**Table 4:** Key findings of included publications from the scoping review which reported factors showing an increased risk of developing colic.

Variable	No. of studies	Risk factor reported (multivariable analysis) and measures of association
Age	Archer 2014 [23]	Younger horses with IFEE than other types of colic (p<0.0001). Age 0–5 at greatest risk
	Cohen 1999 [32]	>10yrs (OR = 1.5, 95% CI = 1.1–2.0, p = 0.015)
	Cohen 1996 [33]	*>8yrs (OR = 1.52, 95% CI = 1.29–1.79, p< 0.0001)
	Hassanpour 2007 [37]	Age 2-10yrs (vs <2yrs) (OR = 3.1, p<0.05)
	Kaneene 1997 [7]	Increasing age in years (OR = 1.05, 95% CI = 1.05–1.44, p = 0.012)
	Patipa 2012 [49]	<1 and ≥21 (OR not calculated because age was included as a quadratic predictor, p = 0.012)
	Tinker 1997 [63]	Age 2–10 years (OR = 2.8, 95% CI = 1.2–6.5, p = 0.02) Age >10 years (OR = 1.6, 95% CI = 0.6–4.2, p = 0.34)
Gender	Suthers 2013 [62]	Increased risk of LCV if mare never foaled compared with males (OR = 4.55, 95% CI = 1.30–15.88, p<0.001) Increased risk of LCV if mare ≥1 foal compared with males (OR = 12.86, 95% CI = 3.16–52.27, p<0.001)
Breed	Cohen 2000 [31]	Arabian or miniature horse breeds at increased risk of enterolithiasis compared with non-surgical group (OR = 4.2, CI = 1.1–16.7, p = 0.04)
	Cohen 1999 [32]	Arabians vs other breeds (OR = 2.1, 95% CI = 1.1–4.0, p = 0.020)
	Cohen 1996 [33]	*Arabs + history of colic (OR = 1.28, 95% CI = 1.07–1.61, p = 0.044)
	Hudson 2001 [41]	Thoroughbred breed (OR = 4.7, 95% CI = 1.5–17.7, p = 0.008)
Foaling	Kaneene 1997 [7]	Foaling during study (OR = 2.55, 95% CI = 1.23–5.30, p = 0.012)
Height	Archer 2008I [6]	Taller horses (OR/cm increase = 1.05, CI = 1.01–1.08, p<0.01)
	Archer 2008U [9]	Taller horses (OR/cm increase = 1.07, CI = 1.01–1.12, p<0.01)
	Suthers 2013 [62]	Increased risk of LCV with increasing height (cm) (OR = 1.06, 95% CI = 1.00–1.12, p = 0.03)
History	Archer 2008I [6]	History of colic in previous 12 months (OR = 4.4, CI = 1.5–12.7, p<0.01)
	Archer 2008U [9]	History of colic in previous 12 months (OR = 5.13, CI = 1.39–18.85, p = 0.01)
	Cohen 1999 [32]	History of previous colic (OR = 3.9, 95% CI = 2.6–5.9, p<0.001)
	Cohen 1996 [33]	*History of abdominal surgery (OR = 3.08, 95% CI = 1.86–5.10, p<0.0001)
	Cohen 1995 [8]	History of previous colic (OR = 5.72, 95% CI = 4.70–6.96, p<0.001) History of abdominal surgery for colic (OR = 5.31, 95% CI = 2.56–10.99, p<0.001)
	Suthers 2013 [62]	Increased risk of LCV with >1 colic episode in the last 12 months (OR = 8.73, 95% CI = 1.78–42.74, p = 0.004)
	Tinker 1997 [63]	History of colic in last 5 years (OR = 3.6, 95% CI = 1.9–6.8, p<0.001)
Behaviour	Archer 2008I [6]	Increased risk of EFE in crib-biting/windsucking horses (OR = 67.3, CI = 15.3–296.5, p<0.01)
	Archer 2008U [9]	Increased risk of EFE in crib-biting/windsucking horses (OR = 71.58, CI = 14.26–359.19, p<0.01)
	Archer 2004 [25]	Increased risk of EFE in crib-biting horses (USA group (OR = 34.7, CI = 6.2–194.6, p<0.001), UK group (OR = 8.2, CI = 4.5–15.1, p<0.001)
	Archer 2004b [26]	Increased risk of EFE in crib-biting/windsucking horses (OR = 7.87, CI = 4.05–15.29, p<0.001)
	Escalona 2014 [36]	Increased risk of history of colic in last 12 months with severity of crib-biting/windsucking behaviour (OR = 1.24, CI = 1.10–1.40, p<0.001)
	Hillyer 2002 [40]	Crib-biting or windsucking (OR = 89.46, CI = 8.98–890.69, p<0.001)
	Salem 2017 [56]	Stereotypic behaviour (OR = 2.0, 95% CI = 1.15–3.5, p = 0.01)
	Scantlebury 2015 [57]	Increased risk of recurrent colic with crib-biting or windsucking (OR = 10.1, 95% CI = 2.5–41.0, p<0.001) Increased risk of recurrent colic with weaving behaviour (OR = 3.9, 95% CI = 1.5–10.1, p = 0.004)
	Scantlebury 2011 [58]	Increased risk of recurrent colic within one year with crib-biting or windsucking (OR = 12.1, 95% CI = 1.4–108.1, p = 0.03)
	Suthers 2013 [62]	Increased risk of LCV if horse noted to quid in last 90 days (OR = 7.77, 95% CI = 1.82–33.15, p = 0.005)
Medication	Scherrer 2016 [59]	Total daily NSAID dose (per 1 mg/kg increase) (OR = 1.98, 95% CI = 1.22–3.21, p = 0.005)
	Senior 2006 [60]	Morphine administration (OR = 4.11, 95% CI = 1.39–12.2, p = 0.01)
	Suthers 2013 [62]	Increased risk of LCV if received medication in last 7 days (excluding anthelmintic) (OR = 6.44, 95% CI = 1.52–27.36, p = 0.01)
Carer	Archer 2008I [6]	Owner/relative/spouse not involved in care (OR = 5.5, 95% CI = 2.27–13.33, p<0.01)
	Escalona 2014 [36]	Duration of ownership (months) (OR = 1.02, 95% CI = 1.01–1.02, p<0.001)
	Suthers 2013 [62]	Increased risk of LCV with ≥3 carers (OR = 11.86, 95% CI = 3.70–38.02, p<0.001)
Housing / Turnout	Archer 2008U [9]	Increased risk of EFE with increased stabling in previous 28 days (OR = 3.70, 95% CI = 1.14–9.70, p<0.01)
	Cohen 2006 [30]	Increased risk of DPJ with pasture grazing compared with other colic (Ref = DPJ horses, OR = 0.28, CI = 0.15–0.55, p = 0.0002) and lame horses (Ref = DPJ horses, OR = 0.25, CI = 0.12–0.54,p = 0.0005)
	Cohen 2000 [31]	Increased risk of enterolithiasis if ≤50% of time spent outdoors compared with non-surgical group (OR = 4.5, CI = 1.4–13.9, p<0.01) and surgical group (OR = 4.0, CI = 1.3–12.2, p = 0.02)
	Cohen 1999 [32]	Change of housing within 2 weeks (OR = 2.3, 95% CI = 1.2–4.1, p≤0.007)
	Cohen 1996 [33]	*Recent change in stabling (OR = 0.76, 95% CI = 0.61–0.96, p = 0.044)
	Escalona 2014 [36]	Crib-biting/windsucking and increased duration of stabling during September-November (OR = 1.04, 95% CI = 1.003–1.08, p = 0.035)
	Hillyer 2002 [40]	Number of hours stabled per day (OR = 1.16, 95% CI = 1.04–1.29, p = 0.008)
	Hudson 2001 [41]	No pasture time or recent (2 weeks) decrease in acreage or pasture time (OR = 3.0, 95% CI = 1.4–6.6, p = 0.007)
	Reeves 1996 [54]	Access to 4 pastures (OR = 2.3, 95% CI = 0.9–6.5) vs 1 pasture
	Suthers 2013 [62]	Increased risk of LCV with increased hours stabled in last 14 days (OR = 5.48, 95% CI = 1.03–29.02, p = 0.04) Increased risk of LCV with change in pasture in last 28 days (OR = 4.50, 95% CI = 1.45–13.92, p = 0.007)
Premises	Suthers 2013 [62]	Increased risk of LCV with increasing number of horses (per horse) (OR = 1.01, 95% CI = 1.00–1.02, p = 0.03)
Feed	Cohen 2006 [30]	Increased risk of DPJ when feeding more total concentrate compared with other colic (Ref = DPJ horses, OR = 0.75, 95% CI = 0.64–0.89, p = 0.001) and lame horses (Ref = DPJ horses, OR = 0.66, 95% CI = 0.53–0.81,p = 0.0001)
	Cohen 2000 [31]	Increased risk of enterolithiasis when fed alfalfa hay compared with non-surgical group (OR = 4.2, 95% CI = 1.3–12.9, p = 0.01) and surgical group (OR = 3.7, 95% CI = 1.2–10.7, p = 0.02)
	Cohen 1999 [32]	Change in batch of hay within 2weeks (OR = 9.8, 95% CI = 1.2–81.5, p<0.05) Change of diet within 2weeks (OR = 5.0, 95% CI = 2.6–9.7, p<0.001)
	Cohen 1996 [33]	*Coastal grass hay (OR = 1.34, 95% CI = 1.06–1.70, p = 0.012)
	Cohen 1995 [8]	Change of diet within 2weeks (OR = 2.21, 95% CI = 1.74–2.79, p<0.001)
	Escalona 2014 [36]	More frequent crib-biting/windsucking whilst eating hay compared with haylage (OR = 2.08, 95% CI 1.20–3.60, p = 0.008)
	Hassanpour 2007 [37]	Changes in concentrate feeding during the year (1 per year, OR = 3.3, p<0.05), (more than 1, OR = 1.8, p<0.05) More than 1 change in hay feeding during the year (OR = 2.4, p<0.05) Feeding high levels of concentrate (> 2.5 kg/day dry matter, OR = 5.2, p<0.05), (> 5 kg/day dry matter, OR = 7.1, p<0.05)
	Hassel 2004 [39]	>70% diet of alfalfa vs ≤70% alfalfa (OR = 10.8, 95% CI = 2.6–44.0, p<0.05)
	Hudson 2001 [41]	Recent (2 weeks) change in a batch of hay (OR = 4.9, 95% CI = 2.1–11.4, p<0.001) Recent (2 weeks) change in type of grain or concentrate fed (OR = 2.6, 95% CI = 0.9–7.2, p = 0.064 Fed hay from round bales (OR = 2.5, 95% CI = 1.1–5.6, p = 0.028) Fed <2.7kg (6lb) oats daily (OR = 5.9, 95% CI = 1.3–22.0, p = 0.009)
	Little 2012 [44]	Increased risk of ilial impaction if fed Coastal Bermuda hay (p<0.05) vs surgical colic group (OR = 2.7, 95% CI = 1.2–6.5) vs medical colic group (OR = 5.7, 95% CI = 2.4–13.6) vs non-colic group (OR = 4.4, 95% CI = 2.1–9.1)
	Reeves, 1996 [54]	Whole grain corn (OR = 3.40, 95% CI = 1.45–7.83)
	Salem 2017 [56]	Feeding ground corn between June-October (OR = 1.65, 95% CI = 1.03–2.6, p = 0.04)
	Scantlebury 2015 [57]	Probiotic in diet (OR = 2.4, 95% CI = 0.99–6.0, p = 0.06)
	Suthers 2013 [62]	Increased risk of LCV if fed hay in last 28 days (OR = 4.64, 95% CI = 1.54–13.98, p = 0.004) Increased risk of LCV if fed sugar-beet in last 28 days (OR = 7.23, 95% CI = 2.13–24.62, p = 0.001) Increased risk of LCV with a change in amount of forage fed in last 7 days (OR = 7.41, 95% CI = 1.32–41.71, p = 0.02)
	Tinker 1997 [63]	Concentrate intake of 2.5-5kg / day (OR = 4.8, 95% CI = 1.4–16.6, p = 0.01) Concentrate intake of >5kg / day (OR = 6.3, 95% CI = 1.8–22.0, p = 0.004) Whole grain fed (OR = 0.4, 95% CI = 0.2–0.8, p = 0.01) 1 change in concentrate amount, type or frequency within 1 year (OR = 3.6, 95% CI = 1.6–5.4, p = <0.001) More than 1 change in concentrate amount, type or frequency within 1 year (OR = 2.2,95% CI = 1.2–4.1, p = 0.02) More than1 change in hay within 1 year (OR = 2.1, 95% CI = 1.2–3.8, p = 0.01)
Water	Kaya 2009 [42]	Decreased water intake (OR = 5.03, 95% CI = 2.1–12.3, p = 0.001)
	Reeves 1996 [54]	No access to water (OR = 2.2, 95% CI = 1.2–4.3)
Exercise	Cohen 1999 [32]	Exercise ≥ once/week (OR = 1.6, 95% CI = 1.2–2.2, p = 0.003) vs pastured horses
	Hillyer 2002 [40]	Recent regular exercise programme with a change in exercise vs no exercise (OR = 9.30, 95% CI = 1.68–51.40, p = 0.011)
	Kaneene 1997 [7]	Showing activity (OR = 2.30, 95% CI = 1.03–5.21, p = 0.04)
Anthelmintic prophylaxis	Cohen 1999 [32]	Horse NOT part of a regular deworming program (OR = 2.2, 95% CI = 1.4–3.3, p<0.001)
	Kaneene, 1997 [7]	Increased number of de-wormings during study (OR = 1.23, 95%CI = 1.05–1.44, p = 0.012)
	Little 2002 [44]	Increased risk of ileal impaction with no access to pyrantel in 3 months prior to admission (p<0.05) vs surgical colic group (OR = 3.1, 95% CI = 1.2–7.7) vs medical colic group (OR = 4.0, 95% CI = 1.6–10.0) vs non-colic group (OR = 3.4, 95%CI = 1.6–7.5)
	Salem 2017 [56]	Anthelmintic administered within last 6 months (OR = 2.1, 95% CI = 1.3–3.3, p<0.003)
Parasites	Back 2013 [27]	Presence of A*noplocephala perfoliata* eggs in faeces (OR = 16.4, CI = 2.03–132.0, p<0.009)
	Boswinkel 2007 [29]	A*noplocephala perfoliata* antibody levels higher in horses with colic compared to controls (p<0.001) ANOVA analysis only
	Leblond 2002 [43]	Parasitic lesions present (OR = 2.39, 95% CI = 1.55–3.68, p = 0.0006)
	Proudman 1998 [51]	Increased risk of spasmodic colic with increasing optical density of ≥0.600epg in coprological analysis (OR = 15.46, 95% CI = 1.99–119.8, p = 0.009)
Transport	Hillyer 2002 [40]	History of transport in previous 24 hours (OR = 17.48, 95% CI = 2.16–141.35, p = 0.007)
Hospitalisation	Patipa 2012 [49]	Hospitalisation time 5–7 days (OR = 11, 95% CI = 1.1–12, p<0.001) or ≥8 days (OR = 11, 95% CI = 3.7–31, p<0.001) vs 1–4 days
	Senior 2006 [60]	Out of hours (17:00–09:00)(OR = 2.97, 95% CI = 1.01–8.78, p = 0.05)
Vaccination	Tinker 1997 [63]	Potomac Horse Fever vaccine during study (OR = 2.0, 95% CI = 1.2–3.6, p = 0.005)
Dental	Salem 2017 [56]	Severe orodental disease (OR = 6.8, 95% CI = 1.9–24.32, p<0.001)
	Scantlebury 2011 [58]	Increased risk of recurrent colic within one year if dental problem known (OR = 5.5, 95% CI = 1.3–23.1, p = 0.02)
Location	Archer 2014 [23]	North West region of UK.
Season	Archer 2014 [23]	The relative risk of IFEE increased over the 10 year study period (p<0.0001) with a seasonal increase between July and November.
	Archer 2006 [24]	Both 6 month and 12 month cyclical patterns for all colics, all medical colics, EFE, EGS, surgically treated and large colon displacement / torsion colic groups. 12 month cyclical pattern for large colon impaction group
	Cohen 1999 [32]	Change in weather within 3 days (OR = 3.2, 95% CI = 2.0–4.9, p<0.001)

*Results extracted from Cohen et al., 1996 are solely from multiple logistic regression analysis of risk factors associated with a history of colic and not from analysis of risk factors for a history of chronic intermittent colic.

OR = Odds Ratio, CI = Confidence Interval, LCV = Large Colon Volvulus IFEE = Idiopathic Focal Eosinophilic Enteritis, EFE = Epiploic Foramen Entrapment, DPJ = Duodenitis-Proximal Jejunitis

### Key findings

There were 22 different risk factors reported as statistically significant from multivariable analyses across the 52 papers. The risk factors identified were categorised into three broad areas: horse-related factors, management-related factors and environment-related factors. The horse related factors were: age; gender; foaling history; breed; height; previous medical history; behaviour; medication. The management related factors were: carer; housing/turnout; premises; feed; water; exercise; anthelmintic prophylaxis; parasites; transport; hospitalisation; vaccination; dental care/disease. The environmental factors were: season; location (Table 4). The details of each factor and the key findings from each area are described in Table 4.

## Results Part 2. Systematic review of management change

### Study selection

**Fig 2:**
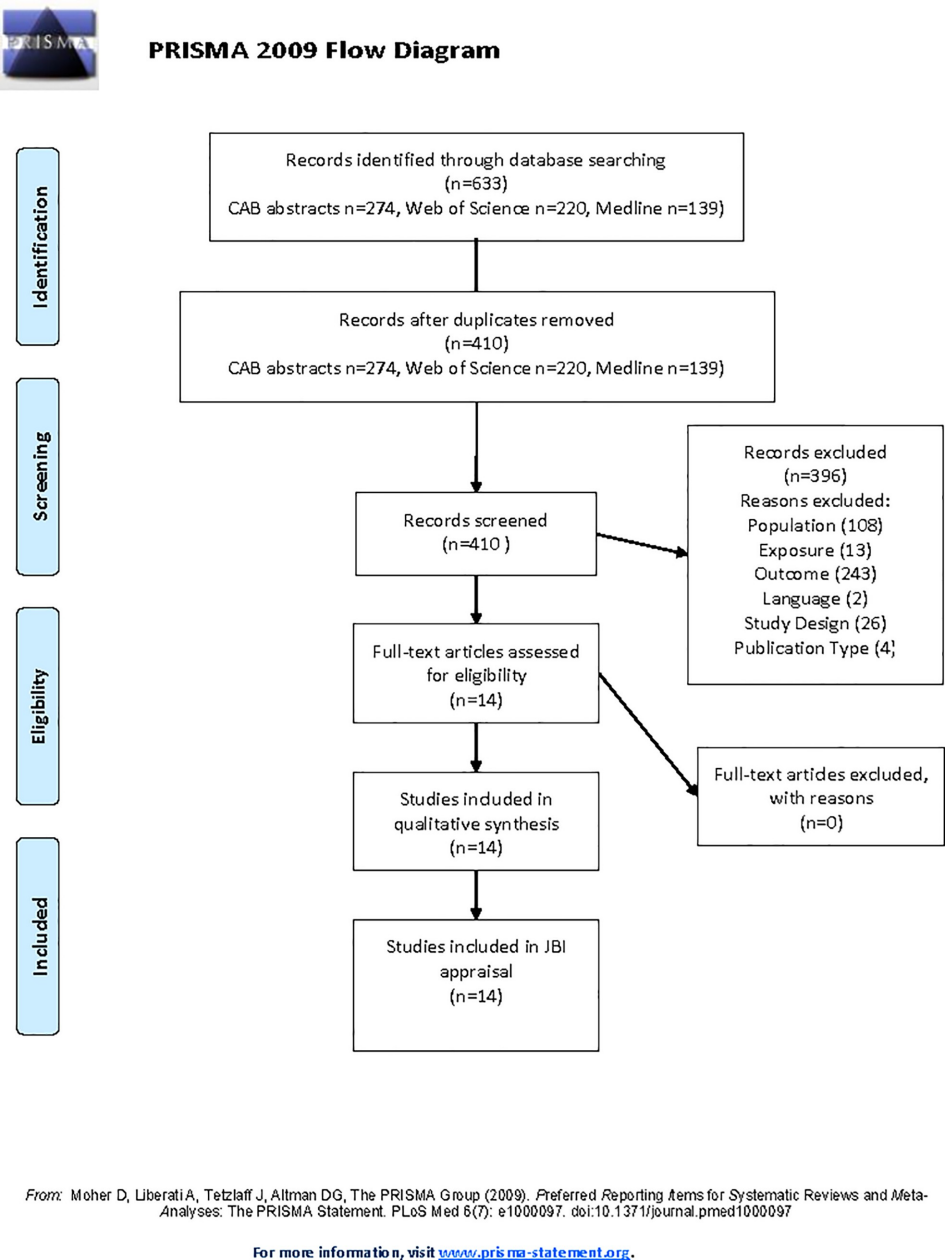
PRISMA 2009 flow diagram for the numbers of studies identified, screened, assessed for eligibility, and included in a systematic review of the risk factors for colic relating to management change.

The initial search identified 633 publications; 410 publications remained following removal of duplicates, and review of the titles, and these abstracts were reviewed again inclusion/exclusion criteria. Full text review and assessment with the JBI critical appraisal tools was performed on 14 publications (Fig 2).

### Study characteristics

The data extracted on study characteristics consisted of the dates of the study, country, source of funding, study design, person making the diagnosis, whether diagnosis was confirmed on surgery/necropsy, sample size, and the management factors that were assessed (Table 5).

**Table 5:** Data extraction- Study characteristics for publications included in a systematic review of management risk factors for colic in the horse.

Author	When study was conducted	Country	Source of funding	Study design*	Colic diagnosis*	Cases confirmed on surgery/ necropsy	Trial sample size (Number with colic)	Management factor assessed
Cohen *et al*. [32]	Mar 1997-Feb 1998	USA	University grant	CC	VS	No	2060 (1030)	Housing, bedding, diet, feeding practices, dental care, anthelmintics, immunisation, activity, changes
Cohen *et al*. [8]	Oct 1991-Dec 1992	USA	No funding declared	CC	VS	No	1642 (821)	Housing, bedding, diet, feeding practices, water sources, weather, dental care, anthelmintics, activity
Cohen and Peloso [33]	Oct 1991-Dec 1992	USA	No funding declared	CC	VS	No	1642 (821)	Housing, feeding practice, recent changes, dental care, anthelmintics, vaccination, activity level
Escalona *et al*. [36]	Unclear	UK	University grant	XS	VS/C	No	367 (130)	Duration of ownership, behaviour, housing, turnout, routine healthcare
Hassanpour *et al*. [37]	Unclear. 5yr study	Iran	No funding declared	XS	Unclear	No	260 (23)	Housing, pasture, type of feedstuffs, nutrition, events
Hillyer *et al*. [19]	Jan-Dec 1997	UK	Equine charity grant	XS	VS/C	Unclear	7757 (509)	Seasonality, premises, age, exercise, parasite control and carer
Hudson *et al*. [41]	Jun 1999-Jun 2000	USA	University grant	CC	VS	Unclear	364 (182)	Feed, pasture, water and anthelmintics
Kaneene *et al*. [7]	Feb 1992-Jan 1993 May 1993-Apr 1994	USA	2 State grants and University grant	XS	VS/C	Some	3175 (62)	Housing, use, feeding, watering, anthelmintics
Kaya *et al*. [42]	Aug 2006-Aug 2007	Austria	No funding declared	CC	VS	Unclear	2743 (366)	Housing, use, feeding, watering, anthelmintics
Malamed *et al*. [45]	Jan 2006- Dec 2008	USA	State funding & private donor contributions	CC	VS	No	574 (347)	Behaviour and temperament
Morris *et al*. [47]	Jan 1987- June 1988	USA	No funding declared	XS	VS	Some	1937 (229)	Feed, recent changes, stocking density, anthelmintics, history
Proudman [53]	1992–1997 Post 5 year follow-up	UK	HBLB funding	CC	VS	Some	279 (179)	Temperature, rainfall, historical events/changes
Reeves *et al*. [54]	Mar 1991– Nov 1991	USA & Canada	Animal charity grant	CC	VS	Unclear	812 (406)	Exercise, housing, environment, nutrition, breeding history, veterinary care, temperament, transport
Tinker *et al*. [63]	Nov 1990- Jan 1991	USA	Breed association grant & equine research funding	PCo	O/C	No	1427 (86)	Employees, feed, water, habitat, pasture, health, housing, use, recent changes

* VS = Veterinary practitioner—physical examination, diagnostic tests, or surgery or necropsy. VS/C = Veterinary practitioner and/or carer of the horse. O/C = Horse owner and/or carer. Co = Cohort, CC = Case-control, XS = Cross-sectional, RCo = Retrospective cohort, PCo = Prospective cohort. HBLB = Horserace Betting Levy Board

The studies were conducted between 1990–2008; the dates of the study were unclear or not provided for two studies. The majority (7/14) were conducted within a 12–14 month period, four studies were less than 12 months duration, two studies were conducted over a five year period, and in one study, information on dates was not provided (Table 5).

The majority of studies were located in the USA (8/14) or UK (3/14). One study was located in the USA and Canada, one in Iran and one in Austria (Table 5).

The most common sources of funding declared were University grant funding (4/14), equine charity funding (3/14), or State funding (2/14). Three studies had more than one source of funding. One study had contributions from a private donor, and one had contributions from a breed association. Five studies did not declare any funding sources (Table 5).

The most common study design was case control (8/14), followed by cross-sectional (5/14), and one was a prospective cohort study (Table 5).

A diagnosis of colic was made by a veterinary practitioner in most studies (9/14), by a veterinary practitioner and/or carer of the horse in three studies, and by the owner/carer in one study. The person making the diagnosis was unclear in one study (Table 5).

The diagnosis was not confirmed on surgery/necropsy in seven studies, was confirmed on surgery/necropsy in some cases in three studies, and this information was unclear or not provided in four studies (Table 5).

The number of horses in the sample populations in the 14 studies ranged from 260–7757, and the number of horses with colic in the 14 studies ranged from 23–1030 (Table 5).

The risk factors related to management change that were assessed in this analysis were feed, carer, exercise, pasture, water and housing (Table 5).

### Participant characteristics

The data extracted on study characteristics consisted of the yard/practice types, the respondent drop-out information, the age, breed and gender of the horses studied, and any additional specific demographic information or exclusions.

The study population was sourced through yards/farms/direct approach to horse-owning population for six studies, primary veterinary practices for four studies, and referral hospitals for four studies (Table 6).

**Table 6:** Data extraction—Participant characteristics for publications included in a systematic review of management risk factors for colic in the horse.

Study	Yard/ Practice information	Respondent drop-out information	Age	Breed/ Type	Gender	Specific demographic information and exclusions
Cohen *et al*. (1999) [32]	Texas multi-practice. No. of yards not provided	Not provided	Colic group median 10yrs (1-41yrs) Control group median 7yrs (1-35yrs)	Quarter horse, Thoroughbred, Arabian, Other breed	Colic group—44% mares, 45% geldings, 11%colts Control group 44% mares, 43% geldings, 13% colts	Horses < 6 months old were excluded
Cohen *et al*. (1995) [8]	Texas multi-practice. No. of yards not provided	Not provided	Colic group median 7yrs (1 month-35yrs) Control group median 6yrs (1 month-32yrs)	Quarterhorse, Thoroughbred, Arabian	Overall 56% males,44% females	
Cohen and Peloso (1996) [33]	Texas multi-practice. No. of yards not provided	Not provided	History of colic group median 9yrs (4 months-32yrs) No history of colic group median 5yrs (1 month-35yrs)	Unclear. Only Arabian discussed	History of colic group– 40% mares, 14% stallions/colts, 46% geldings No history of colic group– 45% mares, 15% stallions/colts, 40% geldings	
Escalona *et al*. (2014) [36]	General UK population. No. of yards not provided	180 non-respondents. 367 horses included out of 370 respondents.	Not provided	Not provided	Not provided	Only horses or ponies with crib-biting/windsucking behaviour included. Horses that had died several months/years prior to study were excluded.
Hassanpour *et al*. (2007) [37] )	Tabriz, 10 farms	Not provided	Median 4yrs	51% Arabian, 33% Crossbreed, 6% Thoroughbred, 10% Kurd	Not provided	
Hillyer *et al*. (2001) [19]	UK Thoroughbred training yards (98 Flat and 108 National Hunt)	113 non-respondents. 279 questionnaires included out of 287 respondents.	Not provided	All Thoroughbreds 90.1% horses in training, 6.5% young/maturing, 3.4% breeding	Not provided	
Hudson *et al*. (2001) [41]	Texas multi-practice. No. of yards not provided	419 cases provided of which 182 matched pairs were included, 55 unmatched horses excluded	Not provided	Quarter horse, Thoroughbred, Arabian, Other breed	Colic group—43.4% mares, 13.2% stallions, 43.4% geldings Control group– 42.3% mares, 7.1% stallions, 50.6% geldings	Horses <1 year old were excluded
Kaneene *et al*. (1997) [7]	Michigan 138 randomly selected yards	Not provided	Colic group mean 10.3yrs Control group mean 8.3yrs	Quarter horse, Standardbred, Thoroughbred, Arabian, Other breed	Colic group– 64.5% mares, 16.1% stallions, 19.4% geldings Control group– 53.9% mares, 11.2% stallions, 30.1% geldings	
Kaya *et al*. (2009) [42]	Vienna, 1 University referral hospital	Not provided	Colic group median 11yrs (3 months-36yrs) Control group median 10yrs (9 months-32yrs)	Warmblood, Thoroughbred, Coldblood, Pony and Mixed-bred	Colic group– 41.2% mares, 10.1% stallions, 48.6% geldings Control group– 49% mares, 17.9% stallions, 33% geldings	
Malamed *et al*. (2010) [45]	California, 1 University referral hospital	1912 non-respondents. 574 respondents included and 316 respondents excluded.	1 -≥ 25yrs	Thoroughbred, Warmblood, Morgan, Arabian, Quarter Horse, Mix, Other breed, Mustang	Colic group– 37% mares, 7.5% stallions, 55.5% geldings Control group– 38.3% mares, 4.6% stallions, 57.1% geldings	Horses < 1 year old were excluded. Horses that were euthanased or died during or after treatment period were excluded.
Morris *et al*. (1989) [47]	Georgia, 1 University referral hospital	Not provided	<1 - >15yrs	12 breeds of horse	45.7% mares, 17.5% stallions, 35.8% geldings	
Proudman (1991) [53]	UK, 1 training and orthopaedic rehabilitation yard for international flat or endurance horses	Not provided	Colic group mean–3yrs Control group mean– 5.6yrs	Thoroughbred and Arab	Not provided	
Reeves *et al*. (1996) [54]	Ontario, New York, Ohio, Pennsylvania, Massachusetts, 5 University referral hospitals	Not provided	Colic group mean 8.5yrs (9 months-30yrs) Control group mean 7.1yrs (7 months-32yrs)	Thoroughbred, Standardbred, Quarter Horse, Arab, Warmblood, Other breed	Colic group– 52% mares, 16% stallions, 32% geldings Control group– 47% mares, 19% stallions, 34% geldings	Horses <6 months old were excluded. A list of specific types of surgical and medical colic was used to exclude cases from the colic group (see paper). Control horses with colic within 4 weeks prior to study or admitted with gastro-intestinal-related complaints were excluded.
Tinker *et al*. (1997) [63]	Virginia, Maryland, 31 randomly selected yards	19 yards declined to enrol/continue. 31 yards included of which 3 provided partial information before exiting the study.	<2 - >10 years	Crossbred, Arab, Quarter Horse, Pony, Other breed, Warmblood, Thoroughbred	44% mares, 13% stallions/colts, 43% geldings	

Nine studies did not provide information on respondent drop-out. For the remaining five studies, this information included the number of non-respondents to questionnaires (3/14 studies), the number of unmatched horses in a case control study (1/14 studies), and the number of yards who declined to participate or only provided partial information (1/14 studies) (Table 6).

The mean or median reported age for horses with colic was most commonly between 7–11 years old (six studies), three studies did not provide data on the age of their population, three studies used age categories/ranges, and two studies reported a mean age of four or less (Table 6).

The breed or type of horses involved was reported in most studies–in one study this information was not provided and in another it was not clear. Eleven of the studies involved more than one breed, and all of these included thoroughbred; one study involved only thoroughbreds (Table 6).

Information on the gender of the horses was not reported in four studies. Nine studies reported data on the percentages of mares, geldings and stallions/colts, and one study reported the percentage of males and females. The percentage of mares/females in the colic populations ranged from 37% to 64.5% (Table 6).

Four studies had specific exclusions relating to age, two of these excluded horses less than one year old, and two excluded horses less than six months of age. One study only included horses with crib-biting / windsucking behaviour. There were specific exclusions relating to horses that had been euthanased or the type of colic in three studies (Table 6).

#### Quality appraisal and risk of bias

One study was assessed using the JBI Critical Appraisal tool for cohort studies. It met all Criteria, except for Criterion 7 (valid and reliable measure of outcome) (Table 7, S2).

**Table 7:** Quality appraisal of 1 cohort, 8 case-control and 5 cross-sectional publications appraised using the JBI quality appraisal tools for publications included in a systematic review of management risk factors for colic in the horse. Criteria descriptors can be found in Supporting Information Item 2 (Systematic Review Protocol).

**Publications**	C1	C2	C3	C4	C5	C6	C7	C8	C9	C10	C11	Yes total
**Cohort studies**												
Tinker *et al*. (1997) [63]	Y	Y	Y	Y	Y	Y	N	Y	Y	Y	Y	10/11
**% of criterion attainment**	100	100	100	100	100	100	0	100	100	100	100	
**Case-control studies**												
Malamed *et al*. (2010) [45]	Y	Y	Y	Y	Y	Y	Y	Y	Y	Y		10/10
Reeves *et al*. (1996) [54]	Y	*NA	Y	Y	Y	Y	Y	Y	Y	Y		9/10
Cohen *et al*. (1995) [8]	Y	Y	Y	N	Y	Y	Y	Y	Y	Y		9/10
Cohen and Peloso (1996) [33]	Y	Y	Y	N	Y	Y	Y	Y	Y	Y		9/10
Cohen *et al*. (1999) [32]	Y	Y	Y	N	Y	Y	Y	Y	Y	Y		9/10
Hudson *et al*. (2010) [41]	Y	Y	Y	N	Y	Y	Y	U	Y	Y		8/10
Kaya *et al*. (2009) [42]	Y	N	Y	U	Y	N	Y	Y	Y	Y		7/10
Proudman (1991) [53]	Y	N	Y	Y	Y	Y	N	Y	Y	N		7/10
**% of criterion attainment**	100	62.5	100	37.5	100	87.5	87.5	87.5	100	87.5		
**Cross-sectional studies**												
Kaneene *et al*. (1997) [7]	Y	Y	Y	Y	Y	Y	N	Y				7/8
Escalona *et al*. (2014) [36]	Y	Y	Y	U	Y	Y	U	Y				6/8
Hillyer *et al*. (2001) [19]	Y	Y	Y	N	Y	Y	N	Y				6/8
Morris et al. (1989) [47]	Y	Y	Y	Y	U	U	Y	U				5/8
Hassanpour et al. (2007) [37]	N	N	U	N	N	N	U	U				0/8
**% of criterion attainment**	80	80	80	40	60	60	20	60				

Y: Yes, N: No, U: Unclear

*Matching was carried out in a pilot study but matching variables were not found to be influential and deemed unnecessary for the main study.

Eight studies were assessed using the JBI Critical Appraisal tool for case-control studies. One study met all ten Criteria, four studies met nine of the ten Criteria, one study met eight and two studies met 7/10. All eight studies met the case-control studies Criteria 1 (groups comparable), 3 (same criteria for cases and controls) and 9 (sufficient duration of exposure). Five studies met Criterion 2 (appropriate matching of cases and controls). Only three of the studies met Criterion 4 (standard, valid and reliable measure of exposure). Seven of the studies met Criteria 6 (identification of confounding factors), 7 (strategies to deal with confounding factors), 8 (standard, valid and reliable assessment of outcomes) and 10 (appropriate statistical analysis) (Table 7, S2).

Five studies were assessed using the JBI Critical Appraisal tool for analytical cross-sectional studies. None of the studies met all the Criteria. One study met seven of the eight Criteria, two met 6/8, one met 5/8, and one study met none of the eight Criteria. Four of the studies met the analytical cross-sectional studies Criteria 1 (inclusion criteria clearly defined), 2 (subjects and setting described in detail) and 3 (valid and reliable measure of exposure). Two of the studies met Criterion 4 (identification of confounding factors). Three of the studies met Criteria 5 (strategies to deal with confounding factors), 6 (participants free of outcome at exposure) and 8 (sufficient duration of follow up time). Only one study met Criterion 7 (valid and reliable assessment of outcomes) (Table 7, S2).

### Synthesis of results

The management risk factors identified from the 14 included publications related to feed, carer, exercise, pasture, water and housing. Eight studies reported an increased risk of colic associated with feed, but the specific factors investigated varied. Change in diet was the most commonly reported risk factor for colic–three studies reported an increased risk with a change in concentrate, four studies reported an increased risk with a change in hay, and two studies reported an increased risk with change in diet. The time period specified for the change varied, with three studies a change within previous two weeks, and two studies specifying a change within one year of the colic episode. Three studies reported an increased risk with feeding concentrate >2.5kg/day or oats >2.7kg/day. Two studies reported an increased risk with feeding whole grain corn, but one study reported this as a decreased risk of colic. One study reported an increased risk of colic with coastal grass hay and one reported an increased risk with feeding hay from round bales. One study reported an increased risk of crib-biting/windsucking with eating hay compared to haylage (Table 8).

**Table 8:** Statistically significant results of included publications from a systematic review of management risk factors for colic in the horse.

Variable	Author	Study design	Risk factor identified (multivariable analysis) and measures of association
Feed	Tinker *et al*. (1997) [63]	Cohort	Concentrate intake of 2.5-5kg / day (OR = 4.8, 95% CI = 1.4–16.6, p = 0.01) Concentrate intake of >5kg / day (OR = 6.3, 95% CI = 1.8–22.0, p = 0.004) Whole grain fed (OR = 0.4, 95% CI = 0.2–0.8, p = 0.01) 1 change in concentrate amount, type or frequency within 1 year (OR = 3.6, 95% CI = 1.6–5.4, p = <0.001) More than 1 change in concentrate amount, type or frequency within 1 year (OR = 2.2,95% CI = 1.2–4.1, p = 0.02) More than1 change in hay within 1 year (OR = 2.1, 95% CI = 1.2–3.8, p = 0.01)
	Cohen *et al*. (1999) [32]	Case-control	Change in batch of hay within 2weeks (OR = 9.8, 95% CI = 1.2–81.5, p<0.05) Change of diet within 2weeks (OR = 5.0, 95% CI = 2.6–9.7, p<0.001)
	Cohen and Peloso (1996) [33]	Case-control	Coastal grass hay (OR = 1.34, 95% CI = 1.06–1.70, p = 0.012)
	Cohen *et al*. (1995) [8]	Case-control	Change of diet within 2weeks (OR = 2.21, 95% CI = 1.74–2.79, p<0.001)
	Reeves *et al*. (1996) [54]	Case-control	Whole grain corn (OR = 3.40, 95% CI = 1.45–7.83)
	Escalona *et al*. (2014) [36]	Cross-sectional	More frequent crib-biting/windsucking whilst eating hay compared with haylage (OR = 2.08, 95% CI 1.20–3.60, p = 0.008)
	Hudson *et al*. (2001) [41]	Case-control	Recent (2 weeks) change in a batch of hay (OR = 4.9, 95% CI = 2.1–11.4, p<0.001) Recent (2 weeks) change in type of grain or concentrate fed (OR = 2.6, 95% CI = 0.9–7.2, p = 0.064 Fed hay from round bales (OR = 2.5, 95% CI = 1.1–5.6, p = 0.028) Fed <2.7kg (6lb) oats daily (OR = 5.9, 95% CI = 1.3–22.0, p = 0.009)
	Hassanpour *et al*. (2007) [37]	Cross-sectional	Changes in concentrate feeding during the year (1 per year, OR = 3.3, p<0.05), (more than 1, OR = 1.8, p<0.05) More than 1 change in hay feeding during the year (OR = 2.4, p<0.05) Feeding high levels of concentrate (> 2.5 kg/day dry matter, OR = 5.2, p<0.05), (> 5 kg/day dry matter, OR = 7.1, p<0.05) Feeding a whole grain with or without other concentrate components **reduced risk** (OR = 0.6, p<0.05)
Carer	Hillyer *et al*. (2001) [19]	Cross-sectional	**Reduced risk** if owner sole carer for the horse (OR = 0.61, 95% CI = 0.35–1.04, p = 0.062)
Exercise	Cohen *et al*. (1999) [32]	Case-control	Exercise ≥ once/week (OR = 1.6, 95% CI = 1.2–2.2, p = 0.003) vs pastured horses
Pasture	Reeves *et al*. (1996) [54]	Case-control	Access to 4 pastures (OR = 2.3, 95% CI = 0.9–6.5) vs 1 pasture
	Hudson *et al*. (2001) [41]	Case-control	No access or recent (2 weeks) decrease in acreage or pasture time (OR = 3.0, 95% CI = 1.4–6.6, p = 0.007)
Water	Reeves *et al*. (1996) [54]	Case-control	No access to water (OR = 2.2, 95% CI = 1.2–4.3)
	Kaya *et al*. (2009) [42]	Case-control	Decreased water intake (OR = 5.025, 95% CI = 2.1–12.3, p = 0.001)
	Kaneene *et al*. (1997) [7]	Cross-sectional	**Reduced risk** providing group water from sources other than tanks, buckets or automatic drinkers (OR = 0.16, 95% CI = 0.03–0.72, p = 0.017)
Housing	Cohen *et al*. (1999) [32]	Case-control	Change of housing within 2 weeks (OR = 2.3, 95% CI = 1.2–4.1, p≤0.007)
	Cohen and Peloso (1996) [33])	Case-control	Recent change in stabling (OR = 0.76, 95% CI = 0.61–0.96, p = 0.044)
	Malamed *et al*. (2010) [45]	Case-control	Change of housing within 1 week (OR = 3.93, 95% CI = 2.64–5.84, p≤0.001)
	Escalona *et al*. (2014) [36]	Cross-sectional	Crib-biting/windsucking and increased duration of stabling during September-November (OR = 1.04, 95% CI = 1.003–1.08, p = 0.035)

A reduced risk of colic was reported in one study if the owner was the sole carer for the horse. An increased risk of colic was reported in one study if the horse was exercised more than once a week, compared to horses at pasture (Table 8).

Risks associated with pasture access were reported in two studies, with one study reporting an increased risk in horses with access to four pastures compared to those with access to one pasture, and the other study reported an increased risk with no access or a recent decrease in pasture access (Table 8).

Risks associated with water access were reported in three studies, with two reporting an increased risk with no or decreased access to water, and one reporting a reduced risk of colic if water was provided from sources other than tanks, buckets or automatic drinkers (Table 8).

A recent change in housing or stabling was reported as associated with an increased risk of colic in three studies, and one study reported an increased risk of crib-biting/windsucking during periods of increased stabling (Table 8).

## Discussion

### Summary

This is the first combined scoping and systematic review in equine veterinary medicine. It is recommended to conduct a scoping review before each systematic review, but most published studies only present the results of the systematic review. The findings of the scoping review are important to establish the breadth and depth of the existing literature, and identify the focus for the final systematic review. In this study, the scoping review provided a broad overview of the current evidence of risk factors across a range of different study types and conditions relating to colic. It summarised the type of study and key findings from 52 publications and 22 different risk factors for colic, which provides a concise source information for veterinary clinicians, researchers and horse owners. The scoping review defined where bodies of evidence for different risk factors were available or lacking. The three main areas of evidence related to horse factors, management factors and environment factors; there was new but limited evidence on factors such as stereotypies and behaviour and owner factors. The scoping review identified management factors as the focus for the systematic review. The systematic review focused on cohort, case-control or cross-sectional studies of management risk factors for colic. Fourteen publications that investigated management factors including feed, carer, exercise, pasture access, water and housing, were appraised. The risk factor identified most frequently was change in management. Change in feeding management was associated with an increased risk of colic in five studies, and a change in housing management was associated with an increased risk of colic in three studies. There were a number of limitations of the current published studies, many of which are common across a range of different veterinary research areas. The systematic review critical appraisal enabled these to be identified and quantified, and were used to inform recommendations for how future studies can be conducted, to improve the quality of evidence.

### Methodology

The purpose of scoping reviews are to map out the existing literature within a specific area, and inform the feasibility and focus of subsequent systematic reviews [15, 18]. Scoping reviews do not appraise the quality of the evidence, but instead provide an overview of the available literature [15]. There are currently three scoping reviews reported in the equine veterinary literature, all published between 2017–2019. These include a scoping review of equine movement/gait analysis [66], a scoping review of systematic reviews and meta-analyses for bovine and equine veterinarians [67], and a scoping review of acupuncture in companion animals [68].

The PRISMA extension for scoping reviews has recently been developed and published [69]. The JBI scoping review protocol is one of the key methodological frameworks currently used, but a range of other approaches have been described. One study [67] did not state which scoping review protocol they used, however they used the AMSTAR tool [70] for assessing the systematic reviews and meta-analyses (which was not applicable to the present study). One study [68] followed the scoping review framework proposed by Arksey *et al*. [15]. The third and most recent study [66] used the JBI scoping review protocol similar to the present study. None of the previous equine scoping reviews published a protocol–Rose et al. (2017) stated that they did not develop a detailed protocol *a priori* to conducting the scoping review, and the other two studies did not provide information on any *a priori* protocols. Development of *a priori* protocol is not mandatory, but helps define the methodology and goals, and reduces reporting bias; publication of protocols can also aid other researchers. Scoping and systematic review protocols can be registered online through Prospero (www.crd.york.ac.uk/prospero), however this is a database of health-related studies funded by the National Institute for Health Research, and their inclusion criteria is studies that are relevant to human health. There are no systems for registration of protocols of veterinary studies that do not have a direct impact on human health.

The data extracted in scoping reviews will vary depending on the objective or PICO (Problem, Intervention, Comparison, Outcome) questions for each scoping review. Scoping reviews may use other methodological frameworks for extracting and assessing data, for example the AMSTAR tool to assess abstracts [67]. The data extracted in this present study followed the recommendations from the JBI scoping review protocol guidelines. The main limitations of a scoping review are the lack of evidence appraisal, and therefore the outcomes are simply a summary of the types of literature available. A subsequent systematic review is required to provide the detailed evidence appraisal. The scoping review is however valuable to inform future research, by identifying gaps in the evidence and highlighting how future research can be improved, as well as identifying areas suitable for systematic review. The present scoping review provides a concise source of information for clinicians of the studies on risk factors, which should provide a useful reference to identify key studies for different areas. The data also highlights the number of studies that have investigated different types of colic, and risk factors. These can be used to inform the feasibility of future systematic reviews, for example on horse age and previous history of colic as risk factors for colic.

The systematic review provides a detailed evidence appraisal, which enables informed decisions on how the information from different studies should be interpreted. The JBI Institute is an international research centre, established in 1996, which has a range of critical appraisal tools and training to enhance evidence-based health care. JBI critical appraisal tools are widely used in systematic reviews [71, 72], and there is a dedicated online journal (JBI Database of Systematic Reviews and Implementation Reports) which publishes systematic reviews which have used the JBI methodology (www.ovid.com/site/catalog/journals/13819.jsp). This present systematic review identified 14 publications for final inclusion and evidence appraisal. The inclusion criteria included cohort, case-control and cross-sectional studies to enable a range of relevant publications to be considered, but each of these study designs have their own critical appraisal tools, and therefore has to be appraised separately. Cohort and case-control studies can be considered more appropriate study designs for assessing risk factors, compared to cross-sectional studies, but this will depend on the methodological quality. A well-planned high quality cross-sectional study may have more reliable results than a poorly conducted cohort study, for example. The results of the quality appraisal in the current systematic review showed that the cohort and case-control studies achieved more of the methodological quality criteria relating to risk of bias in design, conduct and analysis than cross-sectional studies. This aligns with the type of studies best suited to answering an aetiological research question. A prospective cohort study is considered the most appropriate study design (other than systematic reviews and meta-analyses) to answer an aetiological research question [73]. The paucity of cohort studies (1/14) highlights the need for future research and funding to support this and improve the quality of the existing research.

### Limitations

The limitations of both the scoping and systematic reviews were that the ‘grey literature’ was not included, and publications that were not available as full texts or in English were not included. A larger number of databases could have been searched, however those selected were based on the study by Grindlay *et al*. [74], which described which were most appropriate for veterinary journals / publications. Conference proceedings and abstracts were identified through the CAB abstracts searches, but these were not included unless the full paper was available. The published literature may be biased towards positive results. Inclusion of the grey literature (including conference papers, unpublished clinical trials, theses or dissertations) is likely to include more studies with no findings or negative results [75], and therefore publication bias is possible within this study.

Appraisal of publications may be subject to researcher bias, the protocols for both the scoping and systematic reviews in this study included appraisal by two independent researchers and the use of validated appraisal tools to ensure validity and reliability. Advice on the search strategy and methodology was obtained from an experienced information specialist (D. Grindlay).

Neither researcher involved in the search or appraisal received formal training in JBI methodology, and neither had experience as a librarian or information specialist, and this may impact the quality of the search and likelihood of errors [75].

One of the limitations of the scoping and systematic reviews is that colic is defined as abdominal pain, and there are a number of potential different causes. The studies identified varied in terms of whether they investigated specific causes of abdominal pain, or horses showing clinical signs of abdominal pain irrespective of the cause. The scoping review methodology enabled this broad range of literature to be drawn together and categorised, and the charting process identifies the different aims and types of studies. This did however introduce a potential for error or lack of reproducibility, as the decision on whether to include studies depended on the researchers’ interpretation that the study investigated colic. Limiting the review to studies that gave a clear and standardised definition of colic would ensure that the review was rigorous and reproducible by other researchers, however this would also have excluded the majority of studies. In this review, all abstracts and papers were reviewed and agreed by two researchers, with a third researcher contributing if there was disagreement, and any studies which were ambiguous at the title or abstract stage were retained for full evaluation. Recommendations are made below to suggest improvement for future research (e.g. including definitions of key terms such as colic), which would ensure that future reviews could be rigorous and repeatable in their inclusion and appraisal of studies.

There were a number of limitations of the study population used in the studies for both the scoping and the systematic review, which were highlighted through the data analysis. Many studies were not representative of the general population, both in terms of their geographical location and the type of veterinary practices where the data was collected. There was a relatively high proportion of studies based within referral hospitals (25/52 studies in the scoping review and 4/14 studies in the systematic review), which may limit the transferability of findings to the wider horse population. The majority of studies were based solely or partly in the US (22/52 studies in the scoping review and 8/14 studies in the systematic review), and the current study highlights the need for multicentre international studies to determine which risk factors are influenced by geographical location. The majority of studies in the systematic review were conducted more than 18 years ago: 9/14 studies were conducted before 2000, and the only prospective study in the present review was conducted in 1990/1991. There is therefore a need to repeat some of this research to determine whether these findings are still relevant to current equine management systems, particularly in an industry where there have been major changes in approaches to management and nutrition of the horse.

Prospective cohort studies are the most appropriate study design but are expensive and time-consuming to conduct. The most commonly used study design was case-control studies (33/52 studies in the scoping review and 8/14 studies in the systematic review). Case-control studies are appropriate for assessing risk factors, but may be susceptible to sampling bias or confounding factors. Criteria 1, 2, 3, 5 and 8 of the JBI critical appraisal tool for case-control studies relate to the use of controls. This was assessed in the critical appraisal in the systematic review. Controls were comparable to cases in terms of source population and in most publications, appropriate matching was conducted (62.5% of case-control studies). Areas of poor methodological quality across many publications in the systematic review included exposure/risk factor measurement (37.5% adherence in case-control studies) and outcome/colic assessment validity (20% in cross-sectional studies), which were affected by compromised objectivity through observer reporting of colic cases by a variety of sources and the difficulty of confirming a diagnosis in many cases. Many studies did not provide a definition of colic, or clarify whether they included or excluded non-gastrointestinal cases of abdominal pain. A definition of abdominal pain, and how this was defined and determined by the researchers/and or assessors is important to enable comparison between different studies and determine the validity of outcome measures.

Across both the scoping and systematic review, there was significant variation in methodology, and often the justification for selecting risk factors, categorising ranges or selecting reference ranges was not stated, nor was it clear why authors had used different approaches to those described in previous papers. For example, two studies [54, 63] identified feeding whole grain as a potential risk factor; however one [54] did not describe what type of whole grain was investigated and the other [63] specified whole grain corn as the factor of interest. Another example is that the length of time measured between management change factors and occurrence of abdominal pain varied between two weeks [8, 32, 41] and one year [37, 63].

Reference categories that were used for analysis were often inconsistent across different publications, for example age was reported as a risk factor in seven studies in the scoping review, but both the age categories and the reference ranges used varied between studies. Variation in reference ranges, definitions and categories, without giving any justification for alterations, limits the ability to consolidate findings in a comparative review. Consistency across research is essential to demonstrate a valid risk factor.

The time duration of the studies also varied. In the systematic review, most studies were 12 months or more. Four studies were less than 12 months duration [19, 32, 54, 63], but a number of studies were longer duration, sometimes unrelated to the calendar year, and this may introduce a confounding factor. One study [55] for example, was conducted between January 1987 and June 1988, and therefore will have collected two sets of data for the months of January to June. Ideally, study time periods should be based around 12 month intervals (e.g. 12, 24 or 36 months), and time of year and season should be considered as potential confounding factor in data analysis.

There is likely to be an interaction between many risk factors, which may confound or influence results of non-standardised studies. This highlights the importance of multivariable logistic analysis, and also the effect of the researcher in identifying biologically plausible interactions when developing the final model. Most publications failed to acknowledge confounders or factors introducing bias.

There were only two studies [54, 57] which incorporated specific owner factors into their investigation, and yet this is a complex and influential aspect of the care of the horse. The more recent study [57] highlighted the variation in owner attitudes and their approaches to colic and horse management. Factors such as the owner’s experience, the number of horses they care for, and their attitudes towards preventative health care (such as anthelmintic use and dental care), should be considered in future research on risk factors.

The main limitation of this systematic review and much of the evidence-based veterinary medicine across other diseases, is that it is based on less than ideal levels of evidence. Challenges within equine veterinary medicine as a whole are the lack of large scale data collection, the paucity of multi-centre international studies, and the high cost of conducting high quality studies (such as prospective cohort studies for risk factors), and this was demonstrated clearly in the present reviews on colic. The ideal study design is a multi-centre international prospective cohort study that spans different aspects of the horse population, but no studies currently meet these criteria. There have been some successful international collaborations [6], and the development of online tools for recording and exchanging data makes this more achievable. If future studies are designed using a standardised method with consideration of previous research, levels of bias could be minimised, and findings repeated and validated across different studies and populations. Key aspects going forward will be the online publication of methodology and data, and the use of standardised keywords to enable effective electronic searches [76]. Retrospective tagging of keywords to dated publications would aid in collating research and ensuring it is indexed into the correct category.

Key recommendations for future research, arising from the present reviews are:

The establishment of international, multi-centre, prospective cohort studies for investigating risk factors to increase the number and quality of evidence available.

Use of similar reference ranges (e.g. used a standardised period of time to identify management change) and categories (e.g. using the same age, breed or sex reference categories to previous studies) to improve levels of evidence. Alterations in methodology should be justified and have a rational basis (e.g. based on new or emerging evidence).

Publication of methodology detail to describe how exposures and outcomes were assessed (e.g. online supplementary information on how colic was defined, inclusion criteria and how colic was assessed or confirmed).

The development of agreed research keywords used across all online publications to facilitate literature searching, using the model of the MeSH (Medical Subject Heading) thesaurus [76].

These recommendations are based on the present scoping and systematic review of risk factors for colic in the horse. However the issues are present across equine veterinary medicine, and the recommendations are therefore relevant as broad principles for improving the overall quality of evidence-based veterinary medicine.

### Summary of evidence

Despite the issues and limitations, these reviews identified and categorised the current evidence, and can be used to make a number of recommendations.

Increasing age of the horse was identified as a significant risk factor in seven studies in the scoping review. However the studies used a range of methodologies, and most used different ranges and reference categories. Appraisal of this evidence is needed to draw further conclusions about the age categories most at risk, but future research needs to show consistency in methodology to enable evidence to be consolidated.

Similarly, previous history of colic was associated with an increased risk of colic in seven studies in the scoping review, and appraisal of this evidence is warranted. However, again there is variability in how this is measured, with some studies reporting on colic that has occurred in the previous 12 months, one in the previous five years, and others not providing this detail. The majority of studies defined this as being within the previous 12 months, and using this definition for future research will add to the existing evidence base.

Crib biting and windsucking behaviour were reported as having a positive association with an increased risk of equine colic in five studies in the scoping review. These were published between 2004–2014, and this had not been reported in previous studies. Crib biting and windsucking behaviour should be investigated and considered as a potential confounding factor for future research into risk factors for colic.

The main findings of this study related to the management change factors identified in the scoping and systematic reviews. The largest body of evidence related to feeding management, although this spanned a number of different aspects of feeding, and there was again variations in how each was categorised. The detailed analysis within the systematic review enabled these to be described and evaluated. In the systematic review, high concentrate intake (>2.5kg/day) was identified as a risk factor in three of the studies. This is consistent with physiological studies that have shown changes in hindgut flora with increasing levels of carbohydrate feeding [77]. The amount and type of concentrate associated with increased risk however requires further investigation, including the amount of concentrate related to the size of the horse. Changes in feeding management associated with an increased risk of colic were the main finding relating to feed. This include changes in both forage and concentrate, and changes within the previous 2 weeks or the previous 12 months. Despite these variations in methodology and findings, there is still a reasonable body of evidence to support this as being a risk factor–three case-control studies in the systematic review reported an increased risk with recent (within two weeks) changes [8, 32, 41].

The other main management factor related to changes in housing. This was identified as a significant risk factor in three case-control studies in the systematic review, and in all three studies this was reported as a recent (2 weeks or less) change in housing [32, 33, 45]. A change in housing or stabling may also be associated with change in feed and exercise, and therefore there is likely to be interaction between these factors. Change in management has long been anedoctally associated with colic, but the evidence from the systematic review supports this. Avoiding changes, or introducing changes gradually should be a key aspect of preventative management to reduce the risk of colic in the horse.

### Conclusion

The scoping review collated and summarised the current literature on potential risk factors for colic and the systematic review appraised the evidence on management-related risk factors. The existing studies vary significantly in quality and in the methodology used. There is a need for consistency and transparency in study design and methodology, and for future funding of multi-centre international prospective cohort studies to improve the current evidence base. The present study makes recommendations on key steps to improve the quality of future research, based on critical appraisal of the current evidence. The systematic review identified that feeding high levels of concentrate, changes in feeding management, and changes in housing management were associated with increased risk of colic. These are all modifiable risk factors that can be adjusted by the owner/carer. This study is critical in describing the evidence for different risk factors for colic. This enables horse owners/carers and vets to make evidence-based decisions to plan their management and preventative care programmes to reduce the risk of colic, and identifies key areas for educational programmes for horse owners/carers.

## Supporting information

S1 Checklist PRISMA 2009 checklist. (DOC)

S1 Protocol Protocol for scoping review. (DOCX)

S2 Protocol Systematic review protocol. (DOCX)
