# Supplementary material for: Risk factors for acute abdominal pain (colic) in the adult horse: A scoping review of risk factors, and a systematic review of the effect of management-related changes
Source: PLoS One. 2019 Jul 11;14(7):e0219307. doi: 10.1371/journal.pone.0219307 (PMC6622499; doi:10.1371/journal.pone.0219307)
Supplement: S1 Protocol — (DOCX) [file pone.0219307.s002.docx]

# Supporting Information Item 1. Protocol for scoping review.

# Title

Factors implicated in the increased risk of developing acute abdominal pain (colic) in equids – A scoping review

# Introduction

## Rationale

The manifestation of abdominal pain or discomfort (termed colic) is an important hazard to the health of equids around the world

This review aims to systematically map the literature available on factors associated with the development of colic, and present the main themes and characteristics.

## Objectives

1. To examine the extent of available published, peer-reviewed literature on risk factors for colic in horses and ponies through a systematic search of databases.
2. To chart the characteristics and relevant information of included publications in a pre-designed charting form
3. To gather key themes and concepts from the evidence in order to determine the value in conducting systematic review/s in those areas

# Methods

## Protocol and registration

A PRISMA extension for scoping reviews (PRISMA-ScR) is currently under development. This review will be developed using guidance from a methodological framework for scoping reviews proposed by The Joanna Briggs Institute [[1](#_ENREF_1)] in addition to findings by [Tricco, Lillie (2)](#_ENREF_2). This review has not been registered to an existing protocol.

This project was reviewed and approved by the Ethics Committee, School of Veterinary Medicine and Science, University of Nottingham.

## Eligibility criteria

The eligibility criteria are described in Table 1. A new case of abdominal pain was described as such if onset occurred at least seven days after the end of the previous episode [[3](#_ENREF_3)].

A study was included if full text could be obtained from any of the University of Nottingham libraries or e-libraries, through University of Nottingham journal subscriptions, during one of three visits to the British Library, or from free online Open Access.

Table 1: Inclusion and exclusion criteria for a scoping review of risk factors associated with the development of abdominal pain (colic) in horses and ponies

| Criteria | Inclusion | Exclusion |
| --- | --- | --- |
| Population | All types of domesticated equids (horses and ponies) | Donkeys or mules  Non equids  Foals/neonates |
| Exposures | Publications investigating diagnostic test/s in order to identify a potential risk factor for colic | Publications investigating prognostic and/or diagnostic test/s in order to diagnose a disease or clinical sign relating to colic  Studies of treatment/s for colic  Studies seeking to establish pain scores for colic |
| Comparator | No mention of risk factors for colic |  |
| Outcome | Development of any clinical signs of colic / abdominal pain as recognised by owner/carer or veterinary surgeon, irrespective of severity or survival outcome  Abdominal pain relating to diseases of the gastrointestinal tract  Single and recurrent episodes of abdominal pain  Abdominal pain occurring >30 days following abdominal surgery | Abdominal pain arising from non-gastrointestinal causes  Abdominal pain occurring <30 days following abdominal surgery |
| Language | All languages if translation available | Translation not available |
| Study design | Cohort, case-control or cross-sectional studies | Case series, case reports, randomised controlled trials, narrative reviews, textbook chapters |
| Publication type | Peer and non-peer reviewed publications  Research presented in conference proceedings  Studies published post- 1960 | Unable to obtain full study details  Studies published pre-1960 |

## Information Sources

### Databases

- Medline In-Process & Non-Indexed Citations and Ovid MEDLINE: 1946 - present
- CAB Abstracts (Ovid): 1910 – present
- WEB of Science (Core Collection: Citation Indexes): 1950- present

### Search terms

horse*.mp OR equi*.mp OR equus.mp OR exp horse*/ OR exp equi*/ NOT donkey*

AND

Colic.mp OR abdominal pain.mp OR exp abdominal pain/ OR exp colic/

### WEB of Science search terms

TS=(horse* OR equi* OR equus OR exp horse*/ OR exp equi*/) NOT TS=donkey*

AND

TS=(Colic OR abdominal pain OR exp abdominal pain/ OR exp colic/)

## Study Selection

A primary literature search of databases will be conducted using the search terms outlined previously. The results of each search will be downloaded into bibliological software EndNote X6 (Thomson Reuters). Duplicates will be searched for by author, title and reference and the least complete citation of each duplicate will be deleted within EndNote after each database search and extraction has been completed. Publications will then be assessed through three stages: review of titles for suitable publications, review of abstracts against inclusion and exclusion criteria, and review of the full publications. All titles within the EndNote library will be examined, and their abstracts reviewed. Ambiguous titles will be retained for further review at the next stage (review of abstract).

Abstracts from these publications will then independently assessed by two researchers (SF and LC) for agreement with inclusion and exclusion criteria. Any publications which are ambiguous will be retained and reviewed in the next step (review of the full publication). The full text of the final publication will confirm eligibility for this review and move forward to charting of characteristics. See Fig 1 below.

Search terms

CAB Abstracts (n)

Web of Science (n)

MEDLINE (n)

Kept (n) Deleted (n)

(

Kept (n) Deleted (n)

(

Kept (n) Deleted (n)

(

Duplicate titles removed

Duplicate titles removed

Remaining (n)

Remaining (n)

Remaining (n)

TOTAL (n)

(

Risk Factors

(n)

Full text review. Inclusion criteria met.

Excluded:

Population (n)

Exposure (n)

Outcome (n)

Language (n)

Study Design (n)

Publication Type (n)

Abstract review against eligibility criteria. Carried out independently by 2 reviewers. Confirmed after discussion

Charting of results

Fig 1: Study selection process

## Charting Process

The final full publications will be read and characteristics and relevant information with be charted on the form shown in Table 2. This will be carried out independently by one author (LC).

### Chart Items

Table 2: Chart form – publication characteristics

| **Author (Year)** | **Continent** | **Funding** | **Duration of study** | **Aims of study** | **Study population** | **Risk/s investigated** |
| --- | --- | --- | --- | --- | --- | --- |
|  |  |  |  |  |  |  |
|  |  |  |  |  |  |  |
|  |  |  |  |  |  |  |

## Additional analyses

No additional analysis will be conducted. Methodological quality or risk of bias of included studies will not be appraised, consistent with guidance on scoping review conduct [[1](#_ENREF_1), [2](#_ENREF_2)]

## Dissemination

Publication in peer reviewed journal, lay summary in non-peer reviewed media (veterinary news journals and websites).

# References

1. JBI. The Joanna Briggs Institute Reviewers' Manual: 2015 edition / supplement. The University of Adelaide, Australia: 2015.

2. Tricco AC, Lillie E, Zarin W, O’Brien K, Colquhoun H, Kastner M, et al. A scoping review on the conduct and reporting of scoping reviews. BMC Medical Research Methodology. 2016;16(1):15. doi: 10.1186/s12874-016-0116-4.

3. Hillyer MH, Taylor FGR, French NP. A cross-sectional study of colic in horses on Thoroughbred training premises in the British Isles in 1997. Equine Veterinary Journal. 2001;33(4):380-5. doi: <http://dx.doi.org/10.2746/042516401776249499>.
