# Supplementary material for: Risk factors for acute abdominal pain (colic) in the adult horse: A scoping review of risk factors, and a systematic review of the effect of management-related changes
Source: PLoS One. 2019 Jul 11;14(7):e0219307. doi: 10.1371/journal.pone.0219307 (PMC6622499; doi:10.1371/journal.pone.0219307)
Supplement: S2 Protocol — (DOCX) [file pone.0219307.s003.docx]

**Supporting Information Item 2. Systematic Review Protocol.**

**Title**

In equids, does a change in management compared with no change in management increase the risk of developing acute abdominal pain (colic)?

**Introduction**

**Rationale**

The term colic is used to describe abdominal pain or discomfort, and manifests as a result of a disease or disorder of the alimentary system [[1](#_ENREF_1), [2](#_ENREF_2)]. The causes of abdominal pain (colic) in the horse are often multifactorial. Therefore, identifying factors associated with an increased risk of colic is difficult to achieve through a single study [[3](#_ENREF_3)]. There have been many research attempts to identify factors associated with the increased risk of abdominal pain, with many research questions remaining unanswered. Evidence on management-related risk factors can contribute to the veterinary surgeon’s clinical approach and inform strategies on management and prevention of disease.

This review aims to assess the influence of management-related risk factors on the development of abdominal pain in equines by examining the current evidence base publications investigating the influence of management-related factors on the development of abdominal pain in horses and comparing the outcomes in equines that have not been exposed to the same management-related factor.

**Objectives**

1. Identify the published, peer-reviewed literature on management-related factors associated with the risk of developing abdominal pain (colic) in horses and ponies through a systematic search of databases.
2. Critically appraise the quality of the evidence base
3. Evaluate the quality of evidence on management-related factors associated with the risk of developing abdominal pain (colic).
4. Compare the outcome of management-related factors on the development of abdominal pain in horses with outcomes in equines that have not been exposed to the same management-related factor.
5. Summarise the best evidence on management-related risk factors for equine abdominal pain (colic).

**Methods**

**Protocol and registration**

This review will adhere to PRISMA guidelines. This review has not been registered to an existing protocol.

This project was reviewed and approved by the Ethics Committee, School of Veterinary Medicine and Science, University of Nottingham.

**Eligibility criteria**

The eligibility criteria are described in Table 1. A new case of abdominal pain was described as such if onset occurred at least seven days after the end of the previous episode [[4](#_ENREF_4)].

A study was included if full text could be obtained from any of the University of Nottingham libraries or e-libraries, through University of Nottingham journal subscriptions, during one of three visits to the British Library, or from free online Open Access.

Table 1: Inclusion and exclusion criteria for a systematic review of management-related factors associated with the risk of developing abdominal pain (colic) in horses and ponies

| Criteria | Inclusion | Exclusion |
| --- | --- | --- |
| Population | All types of domesticated equids (horses and ponies) | Donkeys or mules  Non equids |
| Exposures | Change in management (feeding frequency and type, housing, pasture access or exercise) in 2 weeks prior to assessment | No mention of management change |
| Comparator | No change in management (feeding frequency and type, housing, pasture access or exercise) in 2 weeks prior to assessment |  |
| Outcome | Development of any clinical signs of colic / abdominal pain as recognised by owner/carer or veterinary surgeon, irrespective of severity or survival outcome  Abdominal pain relating to diseases of the gastrointestinal tract  Single and recurrent episodes of abdominal pain  Abdominal pain occurring >30 days following abdominal surgery | Abdominal pain arising from non-gastrointestinal causes  Publications which related to specific diseases causing clinical signs of abdominal pain for example grass sickness, lipoma or enterolithiasis  Abdominal pain occurring <30 days following abdominal surgery |
| Language | All languages if translation available | Translation not available |
| Study design | Cohort, case-control or cross-sectional studies | Case series, case reports, randomised controlled trials, narrative reviews, textbook chapters |
| Publication type | Peer and non-peer reviewed publications  Research presented in conference proceedings | Unable to obtain full study details |

**Information Sources**

Databases

- Medline In-Process & Non-Indexed Citations and Ovid MEDLINE: 1946 - present
- CAB Abstracts (Ovid): 1910 – present
- WEB of Science (Core Collection: Citation Indexes): 1950- present

**Search terms**

horse*.mp OR equi*.mp OR equus.mp OR exp horse*/ OR exp equi*/ NOT donkey*

AND

Colic.mp OR abdominal pain.mp OR exp abdominal pain/ OR exp colic/

AND

risk*.mp OR risk factor*.mp OR cause*.mp OR odds ratio.mp OR proportional hazard.mp OR protective factor.mp

AND

chang*.mp OR alteration.mp OR management chang*.mp OR owner chang*.mp OR carer change.mp OR increas*.mp OR decreas*.mp

AND

Owner*.mp OR carer*.mp OR vet*.mp OR rider*.mp OR routine*.mp OR food.mp OR feed.mp OR diet*.mp OR supplement*.mp OR meal.mp OR forage.mp OR cereal.mp OR concentrate*.mp OR nutrition*.mp OR grain.mp OR intake.mp OR hay.mp OR water.mp OR exercis*.mp OR work*.mp OR train*.mp OR box rest.mp OR stabl*.mp OR housing OR yard*.mp OR pasture*.mp OR paddock*.mp OR field*.mp OR turnout.mp OR grass.mp OR graz*.mp OR anthelmintic.mp OR worm*.mp OR deworm*.mp OR parasit*.mp OR vaccin*.mp OR dentist*.mp OR dental.mp OR teeth.mp OR tooth.mp OR environment*.mp OR weather.mp OR season*.mp

OR exp food/ OR exp *exercis/ OR exp anthelmintic/ OR exp *worm/ OR exp *parasite/ OR exp *season/ OR exp routine/

**WEB of Science search terms**

TS=(horse* OR equi* OR equus OR exp horse*/ OR exp equi*/) NOT TS=donkey*

AND

TS=(Colic OR abdominal pain OR exp abdominal pain/ OR exp colic/)

AND

TS=(risk* OR risk factor* OR cause* OR odds ratio OR proportional hazard OR protective factor)

AND

TS=(chang* OR alteration OR management chang* OR owner chang* OR carer change OR increas* OR decreas*)

AND

TS=(Owner* OR carer* OR vet* OR rider* OR routine* OR food OR feed OR diet* OR supplement* OR meal.mp OR forage OR cereal OR concentrate* OR nutrition* OR grain OR intake OR hay OR water OR exercis* OR work* OR train* OR box rest OR stabl* OR housing OR yard* OR pasture* OR paddock* OR field* OR turnout OR grass OR graz* OR anthelmintic OR worm* OR deworm* OR parasit* OR vaccin* OR dentist* OR dental OR teeth OR tooth OR environment* OR weather OR season* OR exp food/ OR exp *exercis/ OR exp anthelmintic/ OR exp *worm/ OR exp *parasite/ OR exp *season/ OR exp routine/)

**Study Selection**

A primary literature search of databases will be conducted using the search terms outlined previously. The results of each search will be downloaded into bibliological software EndNote X6 (Thomson Reuters). Duplicates will be searched for by author, title and reference and the least complete citation of each duplicate will be deleted within EndNote after each database search and extraction has been completed. Publications will then be assessed through three stages: review of titles for suitable publications, review of abstracts against inclusion and exclusion criteria, and review of the full publications. All titles within the EndNote library will be examined, and their abstracts reviewed. Ambiguous titles will be retained for further review at the next stage (review of abstract).

Abstracts from these publications will then independently assessed by two researchers (SF and LC) for agreement with inclusion and exclusion criteria. Any publications which are ambiguous will be retained and reviewed in the next step (review of the full publication). The full text of the final publication will confirm eligibility for this review and move forward to data collection and quality appraisal. See Fig 1 below.

Search terms

CAB Abstracts (n)

Web of Science (n)

MEDLINE (n)

Kept (n) Deleted (n)

(

Kept (n) Deleted (n)

(

Kept (n) Deleted (n)

(

Duplicate titles removed

Duplicate titles removed

Remaining (n)

Remaining (n)

Remaining (n)

TOTAL (n)

(

ManagementRisk Factors

(n)

Full text review. Inclusion criteria met.

Excluded:

Population (n)

Exposure (n)

Outcome (n)

Language (n)

Study Design (n)

Publication Type (n)

Abstract review against eligibility criteria. Carried out independently by 2 reviewers. Confirmed after discussion

Data Collection and Quality Appraisal

Fig 1: Study selection process

**Data Collection Process**

The final full publications will be read and methodological features will be extracted on to the data extraction form shown in Table 2. This will be carried out independently by one author (LC).

**Data Items**

Table 2: Data extraction- methodological features

| **Author** | **Study design** | **Colic diagnosis** | **Cases confirmed on surgery/ necropsy** | **Study population** | **Trial sample size** | **Number with colic** | **Risk identified** |
| --- | --- | --- | --- | --- | --- | --- | --- |
| **Sub group: Feeding** | | | | | | | |
|  |  |  |  |  |  |  |  |
| **Sub group: Housing** | | | | | | | |
|  |  |  |  |  |  |  |  |
| **Sub group: Pasture and Exercise** | | | | | | | |
|  |  |  |  |  |  |  |  |

**Summary measures**

Summary measures used by each publication will be recorded. Meta-analysis is not an objective of this review, and results of included publications will not be combined or summarised.

**Quality appraisal and risk of bias**

Cohort and case-control studies will be appraised against the Joanna Briggs Institute Critical Appraisal tools (Tables 3-5).

Table 3: Joanna Briggs Institute Critical Appraisal tool for cohort studies

|  | Yes | No | Unclear | Not applicable |
| --- | --- | --- | --- | --- |
| 1. Were the two groups similar and recruited from the same population? | □ | □ | □ | □ |
| 1. Were the exposures measured similarly to assign people   to both exposed and unexposed groups? | □ | □ | □ | □ |
| 1. Was the exposure measured in a valid and reliable way? | □ | □ | □ | □ |
| 1. Were confounding factors identified? | □ | □ | □ | □ |
| 1. Were strategies to deal with confounding factors stated? | □ | □ | □ | □ |
| 1. Were the groups/participants free of the outcome at the start of the study (or at the moment of exposure)? | □ | □ | □ | □ |
| 1. Were the outcomes measured in a valid and reliable way? | □ | □ | □ | □ |
| 1. Was the follow up time reported and sufficient to be long enough for outcomes to occur? | □ | □ | □ | □ |
| 1. Was follow up complete, and if not, were the reasons to loss to follow up described and explored? | □ | □ | □ | □ |
| 1. Were strategies to address incomplete follow up utilized? | □ | □ | □ | □ |
| 1. Was appropriate statistical analysis used? | □ | □ | □ | □ |

Table 4: Joanna Briggs Institute Critical Appraisal tool for case-control studies

|  | Yes | No | Unclear | Not applicable |
| --- | --- | --- | --- | --- |
| 1. Were the groups comparable other than the presence of disease in cases or the absence of disease in controls? | □ | □ | □ | □ |
| 1. Were cases and controls matched appropriately? | □ | □ | □ | □ |
| 1. Were the same criteria used for identification of cases and controls? | □ | □ | □ | □ |
| 1. Was exposure measured in a standard, valid and reliable way? | □ | □ | □ | □ |
| 1. Was exposure measured in the same way for cases and controls? | □ | □ | □ | □ |
| 1. Were confounding factors identified? | □ | □ | □ | □ |
| 1. Were strategies to deal with confounding factors stated? | □ | □ | □ | □ |
| 1. Were outcomes assessed in a standard, valid and reliable way for cases and controls? | □ | □ | □ | □ |
| 1. Was the exposure period of interest long enough to be meaningful? | □ | □ | □ | □ |
| 1. Was appropriate statistical analysis used? | □ | □ | □ | □ |

Table 5: Joanna Briggs Institute critical appraisal tool for analytical cross-sectional studies

|  | Yes | No | Unclear | Not applicable |
| --- | --- | --- | --- | --- |
| 1. Were the criteria for inclusion in the sample clearly defined? | □ | □ | □ | □ |
| 1. Were the study subjects and the setting described in detail? | □ | □ | □ | □ |
| 1. Was the exposure measured in a valid and reliable way? | □ | □ | □ | □ |
| 1. Were objective, standard criteria used for measurement of the condition? | □ | □ | □ | □ |
| 1. Were confounding factors identified? | □ | □ | □ | □ |
| 1. Were strategies to deal with confounding factors stated? | □ | □ | □ | □ |
| 1. Were the outcomes measured in a valid and reliable way? | □ | □ | □ | □ |
| 1. Was appropriate statistical analysis used? | □ | □ | □ | □ |

Figure 2: JBI-MAStARI critical appraisal tool: Comparable cohort/case control studies Outcomes of Criteria 5) and 8) will refer to the initial diagnosis of abdominal pain and not to the final outcome of each equid.

**Synthesis of results**

**Methodological features**

Meta-analysis of the results is not an objective of this review, as such, the methodological features of all publications will be discussed and an evidence summary will be presented for each study.

Example of evidence summary for an included study (from another study of colic outcomes, summary based on publication ALLEN, K. J., CHRISTLEY, R. M., BIRCHALL, M. A. and FRANKLIN, S. H. (2012), A systematic review of the efficacy of interventions for dynamic intermittent dorsal displacement of the soft palate. Equine Veterinary Journal, 44: 259–266. doi:10.1111/j.2042-3306.2011.00385.x)

| **Archer 2011** | Journal publication |
| --- | --- |
| Study design | Prospective cohort study.  Multicenter, international study with data collected from 15 clinics |
| Participants | 126 horses diagnosed with Epiploic Foramen Entrapment at exploratory laparotomy. 15 horses lost to follow up. |
| Data collected relevant to review | Survival at 1 year, survival at 2 years. Owner/agent contacted 6 monthly to collect survival data. |
| Outcomes  Results: | Data obtained from owners on survival at 1 and 2 years  For all horses undergoing surgery:  Median survival time 397 days  Survival at 1 year = 41%  Survival at 2 years = 26.5%  For horses surviving anaesthesia:  Survival at 1 year =50.6%  Survival at 2 years = 34.3% |
| Main study limitations | Multiple surgeons and surgical techniques likely to have been utilised due to multicentre nature of study. Surgeon factors not discussed.  Large variations within the group on factors known to effect survival e.g. resection performed/not performed, type of resection performed etc.  Some horses non-survivors due to financial constraints rather than morbidity  Anaesthetic protocols not discussed |

**Quality Appraisal**

The quality appraisal score of all publications will be tabulated according to the appraisal tool used. Results will show individual appraisal along with criterion attainment across all publications appraised by the same tool. See table 6.

Table 6: Results table for publications appraised using the JBI Critical Appraisal tools

| **Publications** | C1 | C2 | C3 | C4 | C5 | C6 | C7 | C8 | C9 | C10 | C11 | Yes total |  |
| --- | --- | --- | --- | --- | --- | --- | --- | --- | --- | --- | --- | --- | --- |
| **Cohort studies** | | | | | | | | | | | | | |
|  |  |  |  |  |  |  |  |  |  |  |  |  | |
| **% of criterion attainment** |  |  |  |  |  |  |  |  |  |  |  |  | |
| **Case-control studies** | | | | | | | | | | | | | |
|  |  |  |  |  |  |  |  |  |  |  |  |  | |
| **% of criterion attainment** |  |  |  |  |  |  |  |  |  |  |  |  | |
| **Cross-sectional studies** | | | | | | | | | | | | | |
|  |  |  |  |  |  |  |  |  |  |  |  |  | |
| **% of criterion attainment** |  |  |  |  |  |  |  |  |  |  |  |  | |

**Additional analyses**

No additional analysis will be conducted.

**Dissemination**

Publication in peer reviewed journal, lay summary in non-peer reviewed media (veterinary news journals and websites).

**References**

1. Mehdi S, Mohammad V. A farm-based prospective study of equine colic incidence and associated risk factors. Journal of Equine Veterinary Science. 2006;26(4):171-4. doi: 10.1016/j.jevs.2006.02.008.

2. Robertson SA, Sanchez LC. Treatment of visceral pain in horses. Veterinary Clinics of North America: Equine Practice. 2010;26(3):603-17. doi: 10.1016/j.cveq.2010.08.002.

3. White NA. Colic prevalence, risk factors and prevention. Australian Equine Veterinarian. 2009;28(1):42-9. PubMed PMID: 20103173363.

4. Hillyer MH, Taylor FGR, French NP. A cross-sectional study of colic in horses on Thoroughbred training premises in the British Isles in 1997. Equine Veterinary Journal. 2001;33(4):380-5. doi: <http://dx.doi.org/10.2746/042516401776249499>.

## 
